# Machine learning-based mortality risk assessment in first-episode bipolar disorder: a transdiagnostic external validation study

**DOI:** 10.1016/j.eclinm.2025.103108

**Published:** 2025-02-13

**Authors:** Johannes Lieslehto, Jari Tiihonen, Markku Lähteenvuo, Alexander Kautzky, Aemal Akhtar, Bergný Ármannsdóttir, Stefan Leucht, Christoph U. Correll, Ellenor Mittendorfer-Rutz, Antti Tanskanen, Heidi Taipale

**Affiliations:** a Department of Forensic Psychiatry, University of Eastern Finland, Niuvanniemi Hospital, Kuopio, Finland; b Division of Insurance Medicine, Department of Clinical Neuroscience, Karolinska Institutet, Stockholm, Sweden; c Institute for Molecular Medicine Finland, University of Helsinki, Helsinki, Finland; d Center for Psychiatry Research, Stockholm City Council, Stockholm, Sweden; e University of Eastern Finland, School of Pharmacy, Kuopio, Finland; f Department of Psychiatry and Psychotherapy, School of Medicine and Health, Technical University of Munich, Munich, Germany; g Department of Psychiatry, The Zucker Hillside Hospital, Northwell Health, Glen Oaks, NY, USA; h Department of Psychiatry and Molecular Medicine, Donald and Barbara Zucker School of Medicine at Hofstra/Northwell, Hempstead, NY, USA; i Department of Child and Adolescent Psychiatry, Charité - Universitätsmedizin Berlin, Berlin, Germany; j German Center for Mental Health (DZPG), partner site Berlin, Germany; k German Center of Mental Health (DZPG), partner site Munich-Augsburg, Germany

**Keywords:** Machine learning, Bipolar disorder, Pharmacotherapy

## Abstract

**Background:**

Accurate mortality risk prediction could enhance treatment planning in bipolar disorder, where mortality rates rival those of many cancers. Such prognostic tools are lacking in psychiatry, where assessments typically emphasize immediate suicidality while neglecting long-term mortality risks, and their clinical use is debated. We evaluated the recently developed machine learning model MIRACLE-FEP, initially developed for first-episode psychosis, in predicting all-cause mortality in patients with first-episode bipolar disorder (FEBD), hypothesizing that it would provide accurate risk prediction and guide pharmacotherapy decisions.

**Methods:**

We utilized national register-based cohorts of FEBD patients from Sweden (N = 31,013, followed 2006–2021) and Finland (N = 13,956, followed 1996–2018). We assessed the MIRACLE-FEP model’s performance in predicting all-cause mortality using the area under the receiver operating characteristic curve (AUROC), calibration, and decision curve analysis. Additionally, we conducted a pharmacoepidemiologic analysis to examine the relationship between predicted mortality risk and pharmacotherapy effectiveness.

**Findings:**

MIRACLE-FEP achieved an AUROC = 0.77 (95%CI = 0.73–0.80) for 2-year mortality prediction in Sweden and 0.71 (95%CI = 0.67–0.75) in Finland. For 10-year all-cause mortality prediction, the model demonstrated an AUROC of 0.71 in both cohorts. The model demonstrated relatively good calibration and indicated potential clinical utility in decision curve analysis. Among patients with predicted risk exceeding the observed two-year mortality rate in FEBD, the lowest mortality risk was observed with polytherapy regimens (compared to non-use of antipsychotics or mood stabilizers), including quetiapine and lamotrigine (HR = 0.42, 95%CI = 0.23–0.80) or mood stabilizer polytherapy (HR = 0.47, 95%CI = 0.27–0.82). Conversely, in patients with predicted risk below this threshold, complex pharmacotherapy was not associated with a significant reduction in mortality risk.

**Interpretation:**

MIRACLE-FEP offers a promising approach to predicting long-term mortality risk and could guide proactive treatment decisions, such as targeting combination pharmacotherapy, in FEBD.

**Funding:**

The 10.13039/501100004359Swedish Research Council for Health, Working Life and Welfare, FORTE (2021-01079).

**Research in context**

**Evidence before this study**

Bipolar disorder is associated with high mortality rates comparable to those observed in several cancers. Despite the recognition of increased mortality, accurate prognostic tools to predict long-term mortality in bipolar disorder remain scarce. Psychiatric risk assessments often focus on short-term suicide risk, neglecting broader mortality risks, and clinical guidelines do not unanimously recommend their use. Prediction models have shown promise in enhancing prognostic accuracy in various medical fields but have not been widely applied to predict long-term mortality in first-episode bipolar disorder (FEBD). We searched PubMed and Embase using the terms (‘machine learning’ OR ‘artificial intelligence’ OR ‘prediction model’ OR ‘predictive model’) AND (‘mortality’ OR ‘death’) AND (‘bipolar disorder’), focusing on studies published before August 27, 2024, without language restrictions. We identified one model for predicting one-year risk of suicide death in severe mental health disorders (including bipolar disorder) but found no prior publications on prediction models for all-cause long-term mortality risk in FEBD.

**Added value of this study**

To our knowledge, this is the first study to use machine learning to predict all-cause mortality in FEBD. We externally validated a machine learning model (MIRACLE-FEP), originally developed for first-episode non-affective psychosis, for predicting long-term all-cause mortality in patients with FEBD across two extensive, unselected national cohorts. The model, using clinically available and scalable risk prediction variables, demonstrated robust predictive performance for two-year and ten-year mortality predictions across two nationwide cohorts, comparable to risk models used in other medical fields, such as oncology, without significant signs of bias. Our findings suggest that proactive use of this model in clinical settings could assist in optimizing treatment allocation and reducing unnecessary interventions, potentially improving patient outcomes while minimizing resource use. Also, our pharmacoepidemiological findings suggested that the model may offer insights into targeting combination pharmacotherapies, such as quetiapine and lamotrigine or mood stabilizer polytherapy.

**Implications of all the available evidence**

In the evolving efforts to mitigate mortality risk in bipolar disorder, this study offers valuable insights for clinicians and policymakers in planning the application of machine learning to assess future mortality risk in people with bipolar disorder. Also, the findings could inform the development of guidelines for the implementation and targeting of polypharmacy in the treatment of bipolar disorder. If further validated, the MIRACLE-FEP model could be used to ensure that the most intensive and costly treatments and monitoring are directed toward those at the highest risk of death.

## Introduction

Accurate prognosis is crucial for informed medical decision-making and effective communication of personalized risk to patients. While several fields of medicine have developed risk calculators to enhance prognostic accuracy, psychiatry has lagged, particularly in assessing and predicting long-term mortality risks. This gap is particularly problematic in bipolar disorder, where the mortality risk rivals that of many cancers. Specifically, patients with bipolar disorder experience potential years of life loss of 12.3 years,^1^ compared to 7.6 in prostate cancer.^2^ Current psychiatric mortality risk assessments predominantly focus on immediate death from suicide, neglecting broader and long-term mortality risks. Furthermore, existing guidelines provide conflicting advice on using risk prediction models.^3^^,^^4^ To our knowledge, only one predictive model has been developed and externally validated to predict suicide death within one year following assessment in people with severe mental health disorders, including bipolar disorder.^5^^,^^6^ However, no tools currently exist for predicting long-term all-cause mortality in people with bipolar disorder.

The development of a general mortality risk calculator for bipolar disorder holds the potential to enhance patient outcomes by enabling personalized treatment plans and proactive care strategies. Despite clinical guidelines emphasizing individualized care for people with bipolar disorder,^7^^,^^8^ there are no specific recommendations for risk stratification. This gap may limit clinicians’ capacity to identify high-risk patients who might benefit from intensified monitoring and intervention, thereby impeding effective resource allocation. Furthermore, the lack of risk stratification likely results in reactive rather than proactive treatment decisions. For example, previous studies have shown that pharmacotherapy in bipolar disorder often becomes progressively complex, with multiple medication classes being required in the later stages of bipolar disorder.^9^ However, it remains unclear whether different pharmacotherapies could be more effectively targeted through proactive, risk-based assessments in the early stages of bipolar disorder.

Recently, a machine learning (ML) model for all-cause mortality risk, MIRACLE-FEP, was developed and validated using five easily accessible clinical and sociodemographic variables in 25,542 patients with non-affective first-episode psychosis from Sweden and Finland.^10^ Given the similarities in causes of death (e.g., suicide as a primary cause of death in both disorders) and overlapping risk factors of mortality (e.g., substance use, obesity) between bipolar disorder and non-affective psychosis,^11^, ^12^, ^13^ here we evaluated the utility of this ML model for predicting mortality risk in first-episode bipolar disorder (FEBD). Using two large, independent register-based nationwide cohorts from Sweden and Finland, we hypothesized that MIRACLE-FEP’s predictions would provide accurate mortality risk assessments that outperform alternative clinical evaluations. Additionally, we investigated whether the model could assist in proactively identifying groups with varying effectiveness of different pharmacotherapies on mortality risk, hypothesizing that individuals with higher predicted risk would benefit more from more intensive treatment (e.g., a combination of antipsychotics and mood stabilizers or mood stabilizer polytherapy) than those at lower risk.

## Methods

### Study design and data acquisition

Following the TRIPOD + AI reporting guidelines,^14^ we studied two national register-based cohorts of patients with FEBD (ICD-10: F30–F31) from Sweden and Finland extracted from similar register databases and with identical exclusion criteria (Appendix p2). A pre-study protocol was not prepared. Ethical approval for this research was obtained from the Regional Ethics Board of Stockholm (decision numbers: 2007/762-31 and 2021-06441-02) and the Finnish National Institute for Health and Welfare, the Social Insurance Institution of Finland, Finnish Centre for Pensions and Statistics Finland (permissions THL/5279/14.06.00/2023, 31/522/2019, 19023 and TK-53-569-19). As this study was registry-based and involved no direct contact with participants, informed consent was not required according to the legislation of both countries.

Both cohorts included individuals diagnosed with FEBD in either inpatient or specialized outpatient clinics with a minimum of 2 years of follow-up and a maximum age at the inclusion of 45 years. Patients in the Swedish cohort had registered treatment contacts in Sweden and were followed between July 1, 2006, and December 31, 2021. This cohort was identified from the National Patient Register (inpatient and specialized outpatient care) and the MiDAS Register (disability pensions and sickness absence). The Finnish cohort included individuals diagnosed with FEBD and followed from January 1, 1996, to December 31, 2018. This cohort was identified from the Hospital Discharge Register maintained by the National Institute of Health and Welfare, the sickness absence register from the Social Insurance Institution of Finland, and disability pensions from the Social Insurance Institution of Finland and the Finnish Centre for Pensions.

### Mortality risk prediction

We conducted the statistical analyses on mortality risk prediction in R, version 4.1.1 (R Project for Statistical Computing). The primary outcome of this study was all-cause mortality during the follow-up period; the secondary outcome was cause-specific mortality. We utilized MIRACLE-FEP, a model trained using XGBoost to predict mortality risk, which was developed using data from 20,000 patients with first-episode psychosis (ICD-10: F20–F29) in Sweden, followed from July 1, 2006, to December 31, 2021. Comprehensive data (i.e., no missingness) were available for both cohorts on variables used by the MIRACLE-FEP mortality risk model: the number of substance use comorbidities (i.e., within a year prior to FEBD), duration of the first hospitalization (i.e., based on the length of the first psychiatric hospitalization due to FEBD), age, sex, and prior somatic hospitalizations (i.e., within two years prior to FEBD). MIRACLE-FEP is available online for research purposes: https://johannes-lieslehto.shinyapps.io/miracle-fep/. We estimated the minimum sample size for the external validation analyses of the model to be 3589 patients (Appendix p2) using *pmvalsampsize* package in R. Briefly, the method considers performance metrics regarding discrimination and calibration and their expected confidence intervals when evaluating the minimum sample size.

We assessed MIRACLE-FEP’s performance in predicting two-year and ten-year all-cause mortality and individual causes of death (Appendix, p 3). The model’s discrimination was evaluated using AUROC. Other model discrimination metrics (e.g., sensitivity and specificity) were assessed using several thresholds. The model’s calibration (i.e., alignment between predicted and observed mortality probabilities) was assessed graphically using calibration plots and calculating the Brier score, calibration slope, and intercept (calibration-in-the-large). To evaluate MIRACLE-FEP’s predictions’ fairness, we analyzed discrimination and calibration across different subgroups, including immigration status, sex, and socioeconomic status (Appendix p3).

In parallel with analyzing the MIRACLE-FEP’s predictions, we developed another machine learning model using XGBoost to see if a model developed within the FEBD sample outperforms MIRACLE-FEP. We compared this model to the MIRACLE-FEP solely with the Swedish data within a nested cross-validation framework. The 57 predefined variables (measured at the time of the FEBD diagnosis or 1–2 years prior, Appendix p3) were retrieved from National Patient Register (clinical history), the MiDAS Register (disability pensions and sickness absence), the Prescribed Drug Register (previous pharmaceutical treatments), and the LISA register (demographic variables). A detailed description of the development of the alternative ML model (including used hyperparameters) is provided in Appendix p3. The comparison between this model and the MIRACLE-FEP was conducted using De Long’s test.

We performed decision curve analysis to evaluate the potential clinical utility of the MIRACLE-FEP model in guiding interventions (e.g., closer monitoring or proactive treatment) by estimating the standardized net benefit across risk thresholds ranging from 0 to 5% for 2-year mortality and 0–30% for 10-year mortality. The risk threshold represents the level of risk at which an intervention is considered warranted. The standardized net benefit reflects the impact of decisions made using the model, with higher values indicating greater clinical benefit. Currently, there is no established tool for assessing the all-cause mortality risk of FEBD. For comparative purposes, in addition to “treat all” and “treat none” strategies, we compared the MIRACLE-FEP with substance use disorder (SUD) comorbidity—recognized as the strongest risk factor for all-cause mortality in mental health disorders^15^—and the Manchester Self-Harm Rule (MSHR), which has shown comparable performance to human physicians in assessing suicidality^16^ (Appendix p3).

### Pharmacoepidemiologic analysis

We investigated whether the associations of different pharmacotherapies and mortality risk vary between MIRACLE-FEP’s predictions over the total available follow-up post-FEBD (up to 23 years in the Finnish and 15 years in the Swedish cohort). We conducted the pharmacoepidemiologic comparisons by stratifying the cohorts based on a MIRACLE-FEP prediction cutoff corresponding to the approximate mortality rate observed in both cohorts. The main exposures were individual mood stabilizers (Anatomical Therapeutic Chemical, ATC codes N03AF01, N03AG01, N03AX09, N05AN01) and antipsychotics (N05A, excluding lithium). We also investigated antidepressants (N06A), benzodiazepines, and related compounds (N05BA, N05CD, N05CF). In the analysis of each medication, the reference group was the non-use of that medication (e.g., use of antidepressants versus non-use). However, for antipsychotics and mood stabilizers, each was compared to the non-use of either antipsychotics or mood stabilizers. We obtained the drug usage periods for time-varying exposure by analyzing prescription drug purchases using the PRE2DUP method detailed elsewhere.^17^ Briefly, the PRE2DUP calculates sliding averages of defined daily dosages, drug purchase amounts, and individual drug use patterns and also takes into account hospital stays and medicine stockpiling of drugs. Separately, in both the Swedish and Finnish samples, we conducted between-individual Cox regression analyses yielding Hazard ratios (HR) and 95% Confidence intervals (CI) using SAS version 9.4 for each investigated medication. We adjusted these models for age, gender, duration of first hospitalization due to FEBD diagnosis, concomitant pharmacotherapy, medications used to treat substance use disorders (N07BB, N07BC), and sequence of medications. Finally, we combined the results from both cohorts using a fixed-effect meta-analysis by utilizing the *metafor* package in R.^18^

### Role of the funding

The study’s funders had no role in the design, data collection, analysis, interpretation, or writing.

## Results

### Demographic results

We gathered data on 31,013 patients with FEBD from the Swedish cohort (mean [SD] age, 29.4 [8.3] years; 10,400 [33.5%] men; 2-year mortality, 178 (0.57%); mean [SD] follow-up, 8.41 [3.72] years; individuals on disability pensions or sickness absence at baseline, 2249 [7.25%]) and 13,956 patients from the Finnish cohort (mean [SD] age, 30.8 [8.2] years; 5932 [42.5%] men; 2-year mortality, 166 (1.19%); mean [SD] follow-up, 10.8 [4.96] years; individuals on disability pensions or sickness absence at baseline, 2249 [9.11%]). The ten-year follow-up data in terms of data linkage were available for 11,504 patients (364 [3.16%] deaths) from Sweden and 7305 patients (433 [5.93%] deaths) from Finland. In both cohorts (Table 1), the most frequent cause of two-year death was suicide. Patients who died (vs. did not) within two years post-FEBD were older, were more likely male, diagnosed in inpatient care, had previous suicide attempts, and had a comorbid substance use disorder. In the Swedish cohort, compared to a five-fold larger Swedish, age and sex-matched, general population sample (N = 152,253), we observed a higher risk of all-cause mortality during the available follow-up (HR = 3.88, 95%CI = 3.53–4.27, Appendix p15).

**Table 1:** Clinical and sociodemographic characteristics of the study.

Characteristic	Swedish sample (N = 31,013)			Finnish sample (N = 13,956)		
	Died within two years (N = 178)	Two-year survivors (N = 30,835)	Statistical testing (T/χ^2^/z, P-value)	Died within two years (N = 166)	Two-year survivors (N = 13,790)	Statistical testing (T/χ^2^/z, P-value)
Age, mean (SD)	32.00 (8.31)	29.4 (8.31)	T = 4.14, P < 0.0001	33.60 (8.15)	30.7 (8.16)	T = 4.53, P < 0.0001
Males, N (%)	110 (61.80)	10,290 (33.37)	χ^2^ = 62.89, P < 0.0001	115 (69.28)	5817 (42.18)	χ^2^ = 48.17, P < 0.0001
Education, N (%)^a^			χ^2^ = 2.96, P = 0.23			
Elementary (≤9 years)	39 (21.91)	7327 (23.76)		NA	NA	
High School (10–12 years)	90 (50.56)	13,380 (43.39)				NA
University/College (>12 years)	42 (23.60)	8314 (26.96)		NA	NA	
Any employment (year before FEBD), N (%)	97 (54.49)	6690 (53.44)	χ^2^ = 0.042, P = 0.84	80 (48.19)	9113 (66.08)	χ^2^ = 22.57, P < 0.0001
Individuals on disability pension at baseline, N (%)	13 (7.30)	2236 (7.25)	χ^2^ < 0.001, P = 1.00	32 (19.28)	1240 (8.99)	χ^2^ = 19.72, P < 0.0001
The place of FEBD diagnosis, N (%)			χ^2^ = 81.56, P < 0.0001			χ^2^ = 45.11, P < 0.0001
Inpatient Care	62 (34.83)	3767 (12.22)		73 (43.98)	3019 (21.89)	
Outpatient Care	116 (65.17)	27,068 (87.78)		93 (56.02)	10,771 (78.11)	
Previous Suicide Attempts (within two years prior to FEBD), N (%)	30 (16.85)	1754 (5.69)	χ^2^ = 38.66, P < 0.0001	17 (10.24)	449 (3.26)	χ^2^ = 22.68, P < 0.0001
Any substances Use Comorbidities (within a year prior to FEBD), N (%)	48 (26.97)	2435 (7.90)	χ^2^ = 18.95, P < 0.0001	24 (14.46)	829 (6.01)	χ^2^ = 18.95, P < 0.0001
First medications Post-FEBD, N (%)						
Any antipsychotics	80 (66.85)	7637 (24.77)	χ^2^ = 37.47, P < 0.0001	0 (0)	3241 (23.50)	χ^2^ = 49.51, P < 0.0001
Any mood stabilizers	79 (44.38)	14,935 (48.44)	χ^2^ = 1.01, P = 0.32	37 (22.29)	3401 (24.66)	χ^2^ = 0.38, P = 0.54
Any antipsychotic + mood stabilizer	31 (17.42)	3417 (11.08)	χ^2^ = 6.56, P = 0.01	0 (0)	1096 (7.95)	χ^2^ = 13.24, P = 0.0003
Any antidepressants	114 (64.04)	17,035 (55.25)	χ^2^ = 5.19, P = 0.02	89 (53.61)	6530 (47.35)	χ^2^ = 2.33, P = 0.13
Any benzodiazepine or related	52 (29.21)	4193 (13.60)	χ^2^ = 35.22, P < 0.0001	0 (0)	1621 (11.75)	χ^2^ = 20.95, P < 0.0001
Cause of Death, N (%)						
Suicides	119 (66.85)			89 (53.61)		
Natural deaths	35 (19.66)			42 (25.30)		
Accidents and assaults	24 (13.48)			35 (21.08)		

<5, Less than five individuals; FEP, First-episode Psychosis; NA, Not Available.

a , Information missing for some individuals.

### Prediction performance

**Fig. 1:**
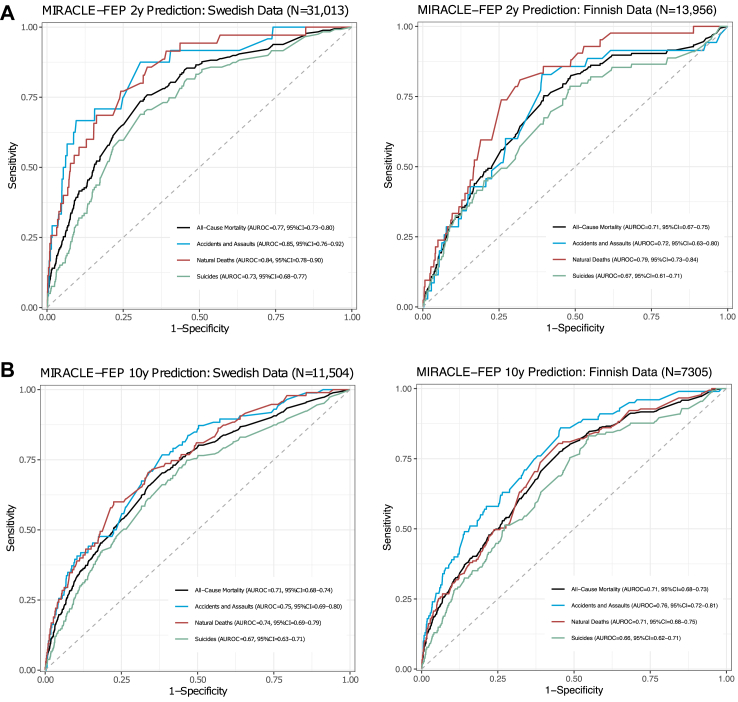
Discrimination of the MIRACLE-FEP model in predicting (A) two-year and (B) ten-year mortality in the Swedish and Finnish cohorts.

In the Swedish cohort, MIRACLE-FEP achieved an AUROC of 0.77 (95% CI = 0.73–0.80) to predict two-year all-cause mortality (Fig. 1a), which did not differ from the predictions made using ML model trained on 57 predictors in the Swedish cohort (AUROC = 0.78, 95%CI = 0.74, 0.81): De Long’s test, D = 0.80, P = 0.422. The AUROC values for MIRACLE-FEP’s predictions of different types of mortality were 0.85 (95% CI 0.76–0.92) for accidental deaths and assaults, 0.84 (95% CI = 0.78–0.90) for natural deaths, and 0.73 (95% CI = 0.68–0.77) for suicides. In the Finnish cohort, MIRACLE-FEP achieved an AUROC of 0.71 (95% CI = 0.67–0.75) for all-cause mortality. The AUROC values for MIRACLE-FEP’s predictions of different types of mortality were 0.79 (95%CI = 0.73–0.84) for natural deaths, AUROC = 0.72 (95%CI = 0.63−0.80) for accidents and assaults, and 0.67 (95% CI = 0.61−0.71) for suicides. In both cohorts, among the two-year survivors, those hospitalized for a suicide attempt or due to bipolar disorder relapse within two years post-FEBD had higher predicted MIRACLE-FEP predictions (Appendix p16).

**Fig. 2:**
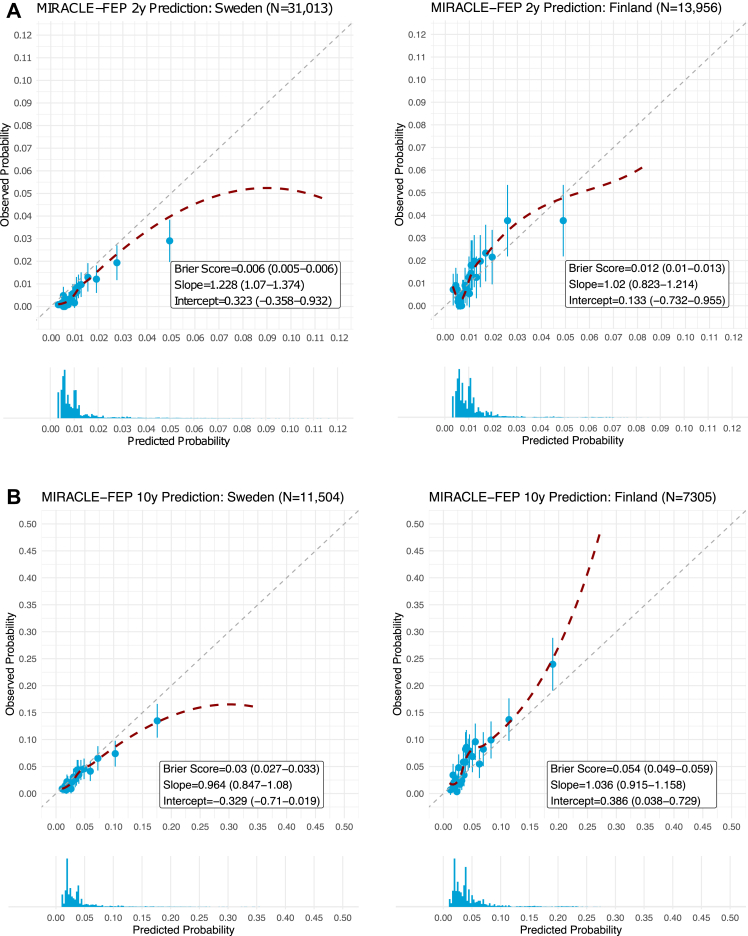
Calibration of the MIRACLE-FEP model in predicting (A) two-year and (B) ten-year mortality in the Swedish and Finnish cohorts. Histograms display the frequency of the model’s probability predictions in a given sample. Red dashed lines represent smoothed nonlinear curves generated using a loess smoother.

MIRACLE-FEP’s 10-year mortality prediction (Fig. 1b) achieved an AUROC = 0.71 (95%CI = 0.68−0.74) for all-cause mortality (accidents and assaults [AUROC = 0.75, 95%CI = 0.69−0.80], natural deaths [AUROC = 0.74, 95%CI = 0.69−0.79], and suicides [AUROC = 0.67, 95%CI = 0.63−0.71]) in the Swedish cohort and AUROC = 0.71 (95%CI = 0.68−0.73) for all-cause mortality (accidents and assaults [AUROC = 0.76, 95%CI = 0.72−0.81], natural deaths [AUROC = 0.71, 95%CI = 0.68−0.75], and suicides [AUROC = 0.66, 95%CI = 0.62−0.71]) in the Finnish cohort. Appendix p17 provides Kaplan–Meier survival curves for all available follow-up periods across both cohorts, with and without stratification by MIRACLE-FEP model predictions. A clear trend is observed in both samples: higher predicted risk is associated with poorer survival outcomes over the total follow-up periods. For both the two- and 10-year predictions (Fig. 2), the model was well calibrated for low probabilities, where most of the patients fell in both samples (>95%). However, at higher predicted probabilities, where very few patients fell (<5%), there was an indication of an overestimation of mortality risk in the Swedish cohort and an underestimation of mortality risk in the Finnish cohort. In the fairness analysis, we did not observe a consistent deviation pattern in the effect of immigration status, sex, or socioeconomic status on discrimination or calibration across both samples (Appendix pp10–11).

### Potential clinical usefulness

**Fig. 3:**
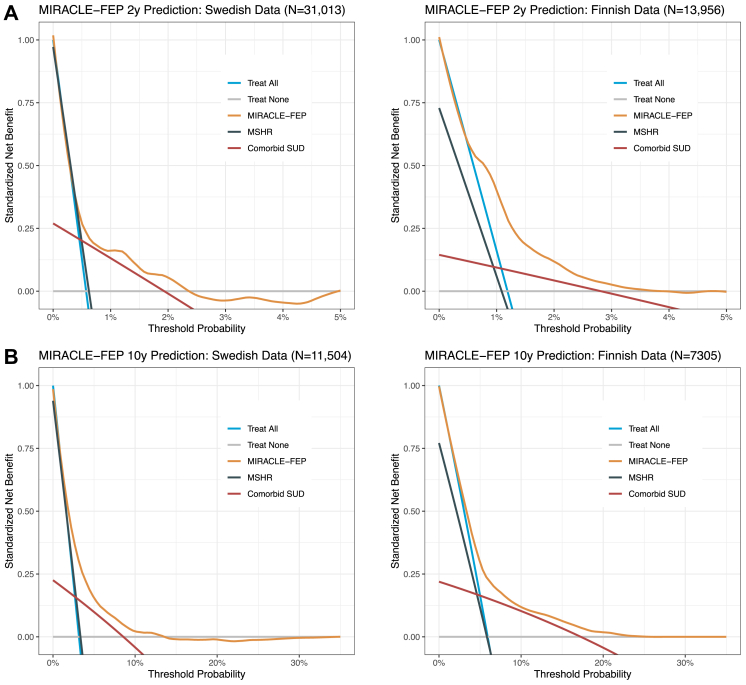
Decision curve analysis of the MIRACLE-FEP model in predicting (A) two-year and (B) ten-year mortality in the Swedish and Finnish cohorts. In classical decision theory, the optimal strategy is the one that maximizes expected clinical utility (net benefit).

In the decision curve analysis for two-year mortality risk predictions, the MIRACLE-FEP showed higher standardized net benefit than treat all, treat none, SUD comorbidity, and the MSHR for a range of threshold probabilities between 0.4%–2.3% in Sweden and 0.6%–3.6% in Finland (Fig. 3a). The same was true for the 10-year risk predictions, being 1.1%–12.9% in the Swedish and 2.3%–24.6% in the Finnish cohort (Fig. 3b).

The MIRACLE-FEP model also demonstrated superior discrimination compared to SUD comorbidity and MSHR for both 2-year and 10-year mortality predictions (Appendix p18): De Long’s tests, all P-values < 0.0001. As detailed in Appendix pp6–9, SUD comorbidity exhibited high specificity but very low sensitivity, whereas MSHR showed high sensitivity but low specificity for predicting 2-year and 10-year mortality risk. MIRACLE-FEP’s performance (e.g., sensitivity and specificity) across a range of risk thresholds is presented in Appendix pp6–9.

We selected thresholds of 1% for 2-year and 3.5% for 10-year mortality predictions since these thresholds provided a balance between sensitivity and specificity (Appendix pp6–9). At MIRACLE-FEP’s 1% threshold for 2-year predictions, sensitivity was 75.84%, specificity was 65.79%, F1 score was 0.025, and Matthews correlation coefficient (MCC) was 0.066 in the Swedish cohort, while sensitivity was 75.90% specificity was 59.67%, F1 score was 0.042, and MCC was 0.076 in the Finnish cohort. Using this threshold corresponded to a standardized net benefit of 15.98% and a potential reduction of interventions (compared to a strategy of treating all patients) by 51.68% in the Swedish cohort and a standardized net benefit of 41.21% and a potential reduction of interventions by 30.59% in the Finnish cohort within that timeframe (Fig. 3, Appendix p19). For MIRACLE-FEP’s 10-year predictions, using a 3.5% threshold corresponded to a standardized net benefit of 27.32% and a potential reduction of interventions (compared to a strategy of treating all patients) by 33.43% in Sweden and a standardized net benefit of 52.08% and a potential reduction of interventions by 15.14% in Finland (Fig. 3, Appendix p19).

### Pharmacotherapy effectiveness

**Fig. 4:**
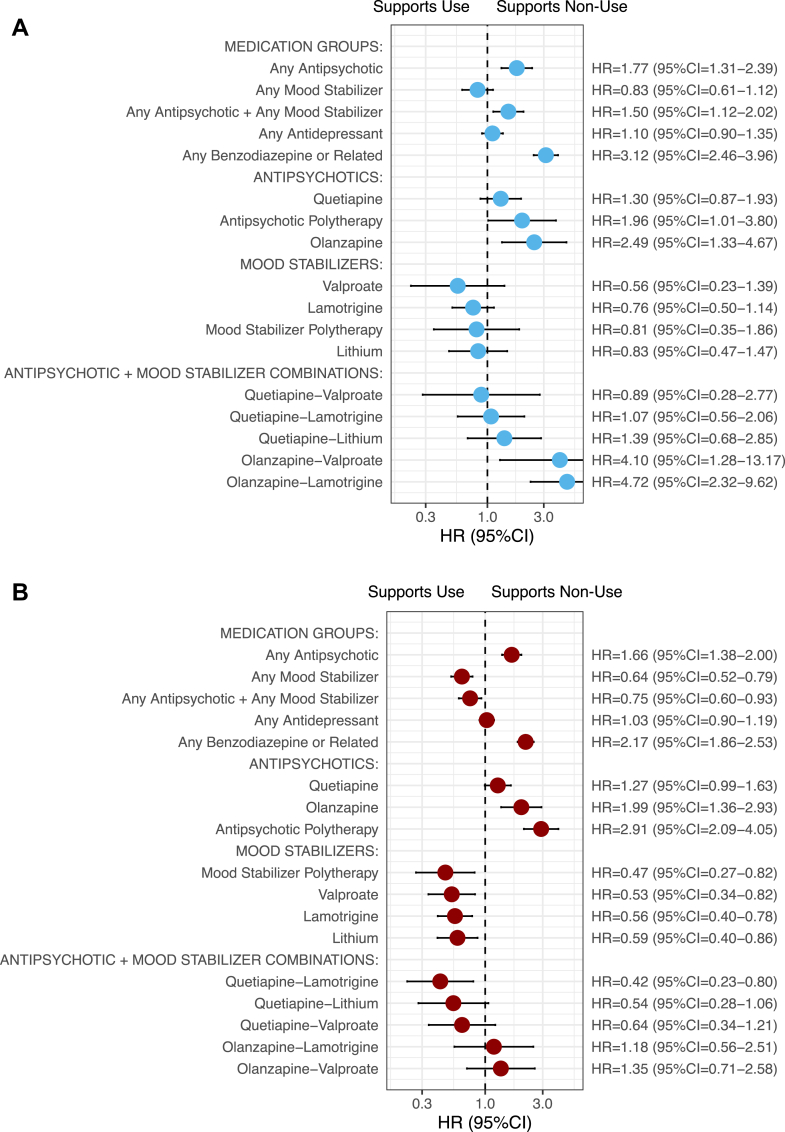
Pharmacoepidemiologic meta-analysis for the association of different pharmacotherapies and the risk of all-cause death in both cohorts. A) Individuals with predicted two-year mortality risk below 1% (63.6%, N = 28,598) and B) individuals with predicted two-year mortality risk above 1% (36.4%, N = 16,371). Each pharmacotherapy was compared with its non-use, except for antipsychotics and mood stabilizers, which were each compared with the non-use of either antipsychotics or mood stabilizers. The analyses of each risk group were adjusted for age, sex, duration of first hospitalization due to FEBD diagnosis, concomitant pharmacotherapy, medications used to treat substance use disorders (N07BB, N07BC), and sequence of medications.

We stratified patients into two groups using MIRACLE-FEP’s two-year risk prediction threshold of 1%. This stratification yielded a pooled sensitivity of 75.9% and specificity of 63.9% across the two samples. Among patients with a predicted mortality risk exceeding 1% (36.4%, N = 16,371), complex pharmacotherapy, including quetiapine and lamotrigine combination therapy (HR = 0.42, 95%CI = 0.23–0.80) and mood stabilizer polytherapy (HR = 0.47, 95%CI = 0.27–0.82), was associated with a significantly lower risk of death compared to non-use of antipsychotics or mood stabilizers, as illustrated in Fig. 4b. In contrast, among patients with a predicted risk below 1% (63.6%, N = 28,598), no pharmacotherapy was significantly associated with reduced mortality post-FEBD (Fig. 4a). However, the lowest mortality risk in this group was observed with mood stabilizer monotherapies, such as valproate (HR = 0.56, 95%CI = 0.23–1.39) and lamotrigine (HR = 0.76, 95%CI = 0.50–1.14). Notably, the combination of antipsychotics and mood stabilizers was associated with an increased risk of death (HR = 1.50, 95%CI = 1.12–2.02) in this group. These results were based on the fixed-effect meta-analysis across both cohorts; the associations of pharmacotherapies and the mortality risk in the individual cohorts are provided in Appendix p20. Lastly, we observed that the likelihood of receiving first-line (i.e., within 30 days post-FEBD) combination treatment with antipsychotics and mood stabilizers was positively associated with the MIRACLE-FEP predicted risk (Appendix pp12–13).

## Discussion

To our knowledge, this is the first study using ML to predict long-term mortality risk in FEBD, which was four-fold compared to the general population. We demonstrated that the MIRACLE-FEP, developed initially using patients with FEP, can stratify long-term mortality risk post-FEBD with performance almost akin to the clinically used risk calculators in predicting prostate cancer progression.^19^ The model demonstrated robust predictive performance without significant evidence of bias related to immigration status, sex, or socioeconomic factors. Decision curve analyses indicated the model’s potential clinical utility. Furthermore, MIRACLE-FEP showed promise in optimizing resource allocation with estimates for reducing unnecessary interventions by 30%–50% over two years and 15%–30% over ten years compared with a scenario where all patients receive the same interventions. Although based on observational data, the pharmacoepidemiologic findings suggested that the model’s predicted risk may help target the early commencement of polypharmacy.

Resource limitations in healthcare necessitate care stratification to ensure that the most intensive and costly treatments and monitoring are directed toward those who benefit the most. However, stratifying patients remains challenging, as unaided clinicians tend to be over-optimistic when predicting low-frequency outcomes, such as suicidal behavior^20^ or mortality.^21^ Various medical fields have developed risk calculators that provide probabilistic predictions to aid clinical decision-making. In contrast, psychiatry has traditionally relied on categorizing patients into broad “high” or “low” risk groups rather than providing probabilistic estimates for outcomes. The reliance on vague categorizations -particularly the uncertainty regarding the actual outcome probabilities within risk groups, compounded by the poor performance of such tools,^22^^,^^23^ has likely contributed to the slower development and adoption of prognostic models in psychiatry. The MIRACLE-FEP model demonstrates the potential for more quantitative risk assessment in psychiatry. The model’s probability estimates demonstrated good calibration within the range where most patients (>95%) fell across both cohorts. However, due to both over-prediction and under-prediction in a small minority of individuals (<5%) at higher probability thresholds, caution is warranted when interpreting higher predicted probabilities. Our findings suggest that it is unlikely that a “general FEBD mortality model” would calibrate perfectly in samples with different mortality rates. Consequently, sample-specific recalibration is advised before clinical model deployment.

Decision curve analyses indicated superior clinical utility of MIRACLE-FEP compared to alternative strategies. In the absence of a fully comparable tool for predicting all-cause mortality in FEBD, we evaluated MIRACLE-FEP against comorbid SUD, the strongest predictor of all-cause mortality in psychiatric disorders,^15^ and the MSHR, a suicide screening tool developed with performance akin to human clinicians.^16^ While it is essential to note that MIRACLE-FEP was developed to predict all-cause mortality, we justified the comparison with MSHR, as suicide is the predominant cause of death in this population.^12^ Nevertheless, future advancements in suicide prediction could benefit from incorporating the model’s estimates alongside additional suicide-specific risk factors. This refinement is particularly relevant, as the MIRACLE-FEP’s predictive accuracy for suicide was consistently lower than for other causes of death.

Previous studies have employed machine learning to predict pharmacotherapy response in bipolar disorder to develop models to personalize treatment based on clinical variables^24^^,^^25^ or neuroimaging.^26^ However, these investigations have been largely constrained by small sample sizes (i.e., typically < 1000 individuals) and should be regarded as proof-of-concept studies, given that none of these studies have produced externally validated ML models. This study aimed to address this gap by testing the externally validated MIRACLE-FEP model, which we used to stratify pharmacoepidemiologic investigations. Our results suggest that individuals with predicted risks above 1% (a third of both cohorts) could potentially benefit from more complex pharmacotherapy regimens. Specifically, combinations such as quetiapine and lamotrigine, as well as polytherapy with mood stabilizers, were associated with over a 50% reduction in mortality risk post-FEBD in this group. Conversely, individuals with lower predicted risks had increased mortality risk when treated with complex regimens, potentially due to the adverse effects of polypharmacy outweighing any mortality benefit in this lower-risk group. Surprisingly, lithium was not the mood stabilizer monotherapy associated with the lowest mortality risk, likely reflecting its limited use as a first-line treatment in FEBD.^27^

A meta-analysis of previous randomized controlled trials has shown that combining mood stabilizers and antipsychotics yields superior efficacy compared with mood stabilizers alone, albeit at the cost of increased side effects, such as somnolence and weight gain.^28^ Thus, the treatment choice should be carefully balanced, ensuring that patients with more severe presentations and a poorer prognosis receive more efficacious regimens while those with a better prognosis are spared unnecessary adverse effects. In the absence of prognostic tools, combination therapy is often introduced reactively, following the failure of monotherapy,^29^ rather than as a proactive therapeutic strategy. Our findings suggest that proactive targeting of combination therapies to mitigate future mortality risks could be an effective strategy. In particular, the combination of quetiapine and lamotrigine, which a randomized controlled trial has previously supported,^30^ appears especially promising in patients with higher predicted mortality risk.

The present study has both strengths and limitations. A key strength lies in using two large, unselected nationwide datasets, comprising approximately 45,000 patients with first-episode bipolar disorder, and using scalable, clinically available risk prediction variables to validate a previously trained ML model externally. As a result, our findings indicate strong generalizability of the MIRACLE-FEP model to similar populations within government-funded healthcare systems. However, it remains to be seen whether the model performs equally well in cohorts drawn from different healthcare systems. Nevertheless, given that we lacked clinician’s assessment of their patients’ mortality risks, it remains unknown how much the model could improve real-world clinical decision-making. The observed association between first-line combination treatment and higher predicted risk suggests that clinicians detect explicitly or implicitly some degree of mortality risk and act to mitigate it, but this requires further investigations. Further, the current model also relies solely on static factors, and future iterations may benefit from incorporating dynamic, treatment-related variables to improve risk prediction over time. Our study is based on observational data. Thus, caution is warranted when interpreting causal relationships between pharmacotherapy and mortality. We lacked access to clinical indications for treatment choices, leaving room for potential confounding by indication despite efforts to mitigate it. Prospective trials must confirm our findings before they can be applied in clinical practice.

The MIRACLE-FEP model offers a novel approach to stratifying long-term mortality risk in FEBD. If further prospectively validated, the model may guide early pharmacotherapy decisions, particularly in targeting complex pharmacotherapeutic regimens for patients at higher mortality risk. Our results can inform more precise treatment guidelines and decision tools, optimizing resource allocation and potentially reducing unnecessary interventions in the treatment of FEBD.

## Contributors

J.L., H.T., and J.T. conceptualized the paper. J.T., M.L., H.T., E.M-R., and A.T. oversaw data collection and project development. J.L, A.T. and HT accessed and verified the data. J.L., H.T., and A.T. were responsible for the statistical analyses. J.L. drafted the manuscript and provided data interpretation. M.L., S.L, CC, A.A., A.K., B.A., and E.M-R. assisted with the data interpretation. All authors participated in finalizing the manuscript, agreed upon the final version of the manuscript and meet the definition of an author, as stated by the International Committee of Medical Journal Editors.

## Data sharing statement

The data used in this study cannot be made publicly available due to privacy regulations. According to the General Data Protection Regulation, the Swedish law SFS 2018:218, the Swedish Data Protection Act, the Swedish Ethical Review Act, and the Public Access to Information and Secrecy Act, these types of sensitive data can only be made available for specific purposes, including research, that meet the criteria for access to this sort of sensitive and confidential data as determined by a legal review. Readers may contact Professor Kristina Alexanderson (kristina.alexanderson@ki.se) regarding the Swedish data. The Finnish data collected for this study are proprietary to the Finnish government agencies, the Social Insurance Institution of Finland, and the National Institute for Health and Welfare, which granted researchers permission and access to data. The data supporting this study’s findings are available from these authorities, but restrictions apply to the availability of these data. The analysis codes used to analyze these data are available upon request to the corresponding author to reproduce the results.

## Declaration of interests

JT, HT, and AT have participated in research projects funded by grants from Janssen-Cilag to their employing institution. JL owns shares for the publicly traded companies Orion, Aiforia, and Optomed. JT has been a consultant and/or advisor to and/or received honoria from Healthcare Global Village, HLS Therapeutics, Janssen-Cilag, Lundbeck, Orion Pharma, Otsuka, Teva, and WebMD Global. HT reports personal fees from Gedeon Richter, Janssen-Cilag, Lundbeck and Otsuka. CUC has been a consultant and/or advisor to or has received honoraria from: AbbVie, Acadia, Adock Ingram, Alkermes, Allergan, Angelini, Aristo, Biogen, Boehringer-Ingelheim, Cardio Diagnostics, Cerevel, CNX Therapeutics, Compass Pathways, Darnitsa, Denovo, Gedeon Richter, Hikma, Holmusk, IntraCellular Therapies, Jamjoom Pharma, Janssen/J&J, Karuna, LB Pharma, Lundbeck, MedAvante-ProPhase, MedInCell, Merck, Mindpax, Mitsubishi Tanabe Pharma, Mylan, Neurocrine, Neurelis, Newron, Noven, Novo Nordisk, Otsuka, Pharmabrain, PPD Biotech, Recordati, Relmada, Reviva, Rovi, Sage, Seqirus, SK Life Science, Sumitomo Pharma America, Sunovion, Sun Pharma, Supernus, Takeda, Teva, Tolmar, Vertex, and Viatris. He provided expert testimony for Janssen and Otsuka. He served on a Data Safety Monitoring Board for Compass Pathways, Denovo, Lundbeck, Relmada, Reviva, Rovi, Supernus, Teva and Xenon. He has received grant support from Janssen and Takeda. He received royalties from UpToDate and is also a stock option holder of Cardio Diagnostics, Kuleon Biosciences, LB Pharma, MindLink, Mindpax, Terran and Quantic. SL has received honoraria as advisor and/or for lectures and/or for educational material from Angelini, Apsen, Boehringer Ingelheim, Janssen, Karuna, Kynexis, Lundbeck, Medscape, Neurotorium, NovoNordisk, Otsuka, Orion pharma, Roche, Rovi, and TEVA. Other co-authors report no conflicts of interest.
