# Supplementary material for: Machine learning-based mortality risk assessment in first-episode bipolar disorder: a transdiagnostic external validation study
Source: eClinicalMedicine. 2025 Feb 13;81:103108. doi: 10.1016/j.eclinm.2025.103108 (PMC11874523; doi:10.1016/j.eclinm.2025.103108)
Supplement: Appendix [file mmc1.pdf]

Supplementary Material

Machine Learning-Based Mortality Risk Assessment in First-Episode Bipolar Disorder: A Transdiagnostic External Validation Study

Supplementary Methods (pp2-3)
Supplementary Tables 1-9 (pp4-13)
Supplementary Figures 1-7 (pp14-20)
Supplementary References (p21)

Supplementary Methods.

1.1. Datasets

We identified the Swedish cohort using data from the National Patient Register (inpatient and specialized outpatient care) and the MiDAS Register (disability pensions and sickness absence). In the Swedish cohort, we utilized the following registers: the National Patient Register (all hospital care periods and specialized outpatient visits with diagnoses from July 2005 to December 2021), the Prescribed Drug Register (prescription drug purchases from July 2005 to December 2021), the Causes of Death Register (causes of death from 2006 to 2021), and the LISA register (demographic characteristics). Flow charts describing the study cohorts are shown in Supplementary Figure 1. We included persons with a first-time diagnosis of bipolar disorder (BD) (no previous diagnosis since 1969) between 2006 and 2021 and without a preceding diagnosis of schizophrenia-spectrum disorder (F20-F29) were included (N=71,417). A one-year washout period for antipsychotic and mood stabilizer use (N05A, N03AF01, N03AG01, N03AX09) starting three months before the BD diagnosis (i.e., washout from T-3 months to T-15 months in relation to diagnosis) was applied, leaving 48,893 individuals. Those diagnosed in inpatient care with a care period of more than 100 days were excluded, leaving 48,733 individuals. Further exclusions were made for those who did not have two years of follow-up in terms of data linkage after BD diagnosis or discharge from the first inpatient stay (N=44,171), those who emigrated before two years of follow-up (N=43,971), those who died during the stay related to their first-time BD diagnosis (N=43,924), and those who received an F2 diagnosis within two years (N=42,529). Finally, an age restriction of 45 years was applied, resulting in a final cohort of 31,013 individuals.

The Finnish cohort was identified from the Hospital Discharge Register maintained by the National Institute of Health and Welfare, the sickness absence register from the Social Insurance Institution of Finland, and disability pensions from the Social Insurance Institution of Finland and the Finnish Centre for Pensions. Data for the Finnish cohort were collected from the Hospital Discharge Register (all hospital care periods with diagnoses from 1972 to 2018), the Prescription Register (reimbursed prescription drug purchases from 1995 to 2018), and the Causes of Death Register from Statistics Finland (1972–2018). For the Finnish cohort, we utilized a previously formed first-episode cohort[1,2]. The main difference with the Swedish cohort is that the washout period was directly one year before diagnosis. Only previous diagnoses of F20 and F25 were not allowed. The initial cohort included 26,395 individuals. Persons with any F2 diagnosis (other than the already excluded F20 and F25) were excluded, leaving 24,413 individuals. Those diagnosed in inpatient care with a care period of more than 100 days were excluded, leaving 24,182 individuals. Further exclusions were made for those who did not have two years of follow-up in terms of data linkage after BD diagnosis or discharge from the first inpatient stay (N=21,971), those who died during the stay related to their first-time BD diagnosis (N=21,935), and those who received an F2 diagnosis within two years (N=21,246). Finally, an age restriction of 45 years was applied, resulting in a final cohort of 13,956 individuals.

1.2. Classification of the Causes of Death

We used ICD-10 coding to classify all deaths into external causes (V00-Y99) and natural causes (all other codes). Suicides were identified using codes X60-X84, supplemented by diagnoses with undetermined intent (ICD-10 codes Y10-Y34) to address the potential underreporting of suicides. The remaining external causes included accidents (V01-X59) and assaults (X85-Y09).

1.3. Sample Size Calculation

To determine the minimum sample size required for external validation of the MIRACLE-FEP prediction model, we used the pmvalsampsize package in R (version 0.1.0). This package applies the sample size criteria described by Archer et al. (2020)[3] and Riley et al. (2021)[4], based on key performance metrics. We assumed a two-year mortality incidence of 1.7% in the target population, consistent with rates reported in first-episode non-affective psychosis[5]. The anticipated model discrimination, represented by the area under the receiver operating characteristic curve (AUROC), was set at 0.7, in line with the previous findings[5]. The distribution of predicted probabilities was simulated using a beta distribution, with shape parameters ($\alpha = 1.13$, $\beta = 64.82$) derived from the model's internal validation data in the earlier study by Lieslehto et al. (2024)[5]. Given the rarity of the outcome, we relaxed the target confidence interval widths to 0.25 for the AUROC and 0.65 for both the calibration slope and the observed/expected ratio. Using these criteria, we reached the minimum sample size required for the model validation, which was 3589 individuals.

1.4. Machine Learning Pipeline

We employed XGBoost (https://xgboost.ai/about) to train an alternative machine learning model in the Swedish cohort, utilizing 57 available variables (Supplementary Table 1). No variable included in the model had more than 20% missing data, and no participant had more than 40% missing data. Missing values were not imputed, as XGBoost inherently handles missing data. Model training was conducted within a nested cross-validation framework consisting of 10 outer folds (each with 5 permutations) and 3 inner folds. Hyperparameters were selected based on the original model's development[5], including *eta*/learning_rate (2^{-8}, 2^{-6}, 2^{-4}, 2^{-2}), *gamma* (2^{-16}, 2^{-6}, 2^{2}), *max_depth* (3, 5, 7), *nrounds* (100, 300, 500), and *min_child_weight* (2^{-16}, 2^{-6}, 2^{4}, 2^{8}). Hyperparameter optimization was performed using grid search, with maximal balanced accuracy (mean of sensitivity and specificity) as the selection criterion within the nested cross-validation framework. *Subsample* and *colsample_bytree* were fixed at 0.5. Additionally, we adjusted *scale_pos_weight* variable to the ratio of deaths to survivors in the discovery cohort to account for class imbalance. Other hyperparameters remained at default settings. The results from the nested cross-validation were compared to the model predictions from MIRACLE-FEP using De Long's test.

1.5. Clinical Usefulness Comparison to Alternative Assessments

We conducted a decision curve analysis to evaluate the clinical utility of the MIRACLE-FEP model in predicting all-cause mortality in individuals with FEBD, for which no established risk assessment tool currently exists. For comparative purposes, we assessed MIRACLE-FEP alongside the "treat all" and "treat none" approaches, as well as two predictive markers: comorbidity with substance use disorder (SUD), which is recognized as the strongest predictor of all-cause mortality in mental health conditions, and the Manchester Self-Harm Rule (MSHR). MSHR has demonstrated comparable performance to clinical assessments by physicians in evaluating suicide risk and consists of four core criteria that were slightly modified in the present study: a history of self-harm, previous psychiatric treatment (defined here as psychiatric hospitalization prior to FEBD), use of benzodiazepines in the self-harm attempt (extended in this study to include all poisoning methods), and current psychiatric treatment (defined here as an outpatient visit within three months after FEBD onset or use of psychotropic medications within 30 days of FEBD onset).

1.6. Fairness Analysis

We assessed algorithmic fairness, defined as bias in the predictive model's performance across vulnerable subgroups, by evaluating the MIRACLE-FEP performance metrics (AUROC, Brier score, slope, and intercept) across immigration status (available only in the Swedish cohort), gender, and socioeconomic status (based on median income during the year preceding FEBD). Statistical comparisons between subgroups were made by contrasting the observed performance differences (e.g., AUROC differences between males and females) with predictions generated from 1,000 random permutations of the subgroup labels.

Supplementary Table 1. Variables used for training an alternative machine learning model to predict all-cause mortality within two years post-FEBD and their corresponding importance (gain) to the model.

Variable Name	Importance (Gain)
Male gender (yes/no)	0.169
The number of visits to non-psychiatric hospital in the two years before the FEBD	0.103
Days of the first inpatient stay for those who received their FEBD diagnosis from inpatient care (zero for others)	0.085
The number of different substance use comorbidities (ICD-10: F10-19) year before the FEBD	0.080
Age at FEBD (years)	0.058
Income from work during a previous calendar year before FEBD (brutto, as kronor)	0.056
Diagnosis of FEBD during inpatient visit (yes/no)	0.053
Olanzapine treatment during 30 days after FEBD (yes/no)	0.050
Whether a person has visited a psychiatric hospital within a year before the FEBD	0.047
Benzodiazepines during 30 days after FEBD	0.042
Treatment with antipsychotics during 30 days after FEBD (yes/no)	0.040
Treatment with mirtazapine during 30 days after FEBD (yes/no)	0.039
The sum of unemployment days during a previous calendar year before FEBD	0.038
The number of continuous days on sick leave a year before FEBD	0.029
Treatment with benzodiazepine-related hypnotics (“Z drugs”) during 30 days after FEBD (yes/no)	0.027
Family situation: Single without children (yes/no)	0.025
severity of major depressive disorder one year before FEBD	0.015
Treatment with non-benzodiazepine hypnotics during 30 days after FEBD (yes/no)	0.013
Treatment with SNRI during 30 days after FEBD (yes/no)	0.012
Treatment with SSRI during 30 days after FEBD (yes/no)	0.012
Antidepressants during 30 days after FEBD (yes/no)	0.012
Bipolar disorder with depression	0.012
Residence: rural areas (yes/no)	0.012
Family situation: married or cohabitant with children	0.011
Education: high-school level (i.e., 10-12 years) education (yes/no)	0.010
Family situation: youth (<=20 years) living at home (with their parents) (yes/no)	0.010
Residence: towns and suburbs (yes/no)	0.010
Treatment with non-benzodiazepine anxiolytics during 30 days after FEBD (yes/no)	0.010
Mood stabilizer treatment during 30 days after FEBD	0.009
Suicide attempt (ICD-10: X60-X84, Y10-Y34) year before FEBD	0.009
Education: only elementary level (i.e., 9 years) education (yes/no)	0.008
Education: university/college (i.e., >12 years) education (yes/no)	0.008
Born in Sweden (yes/no)	0.008
Personality disorder (ICD-10: F60-F69) comorbidity year before FEBD (yes/no)	0.007
Family situation: single with children (yes/no)	0.007
On disability pension at baseline	0.005
Residence: densely populated city (yes/no)	0.004
SUD drug use during 30 days after FEBD	0.004

Any employment during a previous calendar year before FEBD (yes/no)	0.004
Treatment with other antidepressants during 30 days after FEBD (yes/no)	0.003
Family situation: married or cohabitant without children	0.003
Treatment with valproate during 30 days after FEBD (yes/no)	0.003
Quetiapine treatment during 30 days after FEBD (yes/no)	0.003
Symptoms of bipolar disorder in remission	0.002
Born outside of Europe (yes/no)	0.002
Treatment with lithium during 30 days after FEBD (yes/no)	0.002
Born in Europe (outside of Sweden) (yes/no)	0.001
ADHD drug use during 30 days after FEBD (yes/no)	0.001
Currently mixed bipolar episode	0.001
Current episode manic/hypomanic without psychotic features	0.001
The degree of depression	0.001
Treatment with other oral antipsychotics during 30 days after FEBD (yes/no)	0.001
Treatment with tricyclic antidepressants during 30 days after FEBD (yes/no)	0.001
Aripiprazole during 30 days after FEBD	0.001
Treatment with risperidone during 30 days after FEBD (yes/no)	0.000
Current episode manic severe with psychotic features	0.000
Substance-induced psychosis year before FEBD	0.000

Supplementary Table 2. Classification performances for the prediction of two-year mortality across MIRACLE-FEP predictions with a range of threshold levels, comorbid SUD, and Manchester Self Harm Rule in the Swedish sample (N=31,013).

Predictor	TP (%)	TN (%)	FP (%)	FN (%)	Sens%	Spec%	BAC%	PPV%	NPV%	LR+	LR-
MIRACLE-FEP											
0.5%	174 (0.56)	4641 (14.96)	26,194 (84.46)	4 (0.01)	97.75	15.05	56.40	0.66	99.91	1.15	0.15
1%	135 (0.44)	20,286 (65.41)	10,549 (34.01)	43 (0.14)	75.84	65.79	70.82	1.26	99.79	2.22	0.37
1.5%	82 (0.26)	26,415 (85.17)	4420 (14.25)	96 (0.31)	46.07	85.67	65.87	1.82	99.64	3.21	0.63
2%	65 (0.21)	28,130 (90.7)	2705 (8.72)	113 (0.36)	36.52	91.23	63.87	2.35	99.60	4.16	0.70
2.5%	51 (0.16)	28,739 (92.67)	2096 (6.76)	127 (0.41)	28.65	93.20	60.93	2.38	99.56	4.22	0.77
3%	43 (0.14)	29,203 (94.16)	1632 (5.26)	135 (0.44)	24.16	94.71	59.43	2.57	99.54	4.56	0.80
3.5%	33 (0.11)	29,786 (96.04)	1049 (3.38)	145 (0.47)	18.54	96.60	57.57	3.05	99.52	5.45	0.84
4%	27 (0.09)	29,981 (96.67)	854 (2.75)	151 (0.49)	15.17	97.23	56.20	3.06	99.50	5.48	0.87
4.5%	25 (0.08)	30,172 (97.29)	663 (2.14)	153 (0.49)	14.04	97.85	55.95	3.63	99.50	6.53	0.88
5%	24 (0.08)	30,385 (97.98)	450 (1.45)	154 (0.5)	13.48	98.54	56.01	5.06	99.50	9.24	0.88
Comorbid SUD	48 (0.15)	28,400 (91.57)	2435 (7.85)	130 (0.42)	26.97	92.10	59.53	1.93	99.54	3.41	0.79
MSHR	173 (0.56)	3229 (10.41)	27,606 (89.01)	5 (0.02)	97.19	10.47	53.83	0.62	99.85	1.09	0.27

Abbreviations: SUD=substance use disorder, MSHR=Manchester Self Harm Rule, TP=true positive, TN=true negative, FP=false positive, FN=false negative, Sens%=sensitivity%, Spec%=specificity%, BAC%=balanced accuracy, PPV%=positive predictive value%, NPV%=negative predictive value%, LR+=positive likelihood ratio, LR-=negative likelihood ratio.

Supplementary Table 3. Classification performances for the prediction of two-year mortality across MIRACLE-FEP predictions with a range of threshold levels, comorbid SUD, and Manchester Self Harm Rule in the Finnish sample (N=13,956).

**Predictor**	**TP (%)**	**TN (%)**	**FP (%)**	**FN (%)**	**Sens%**	**Spec%**	**BAC%**	**PPV%**	**NPV%**	**LR+**	**LR-**
MIRACLE-FEP											
0.5%	154 (1.1)	1647 (11.8)	12,143 (87.01)	12 (0.09)	92.77	11.94	52.36	1.25	99.28	1.05	0.61
1%	126 (0.9)	8229 (58.96)	5561 (39.85)	40 (0.29)	75.90	59.67	67.79	2.22	99.52	1.88	0.40
1.5%	64 (0.46)	11,752 (84.21)	2038 (14.6)	102 (0.73)	38.55	85.22	61.89	3.04	99.14	2.61	0.72
2%	43 (0.31)	12,717 (91.12)	1073 (7.69)	123 (0.88)	25.90	92.22	59.06	3.85	99.04	3.33	0.80
2.5%	27 (0.19)	13,101 (93.87)	689 (4.94)	139 (1.0)	16.27	95.00	55.63	3.77	98.95	3.26	0.88
3%	21 (0.15)	13,246 (94.91)	544 (3.9)	145 (1.04)	12.65	96.06	54.35	3.72	98.92	3.21	0.91
3.5%	14 (0.1)	13,444 (96.33)	346 (2.48)	152 (1.09)	8.43	97.49	52.96	3.89	98.88	3.36	0.94
4%	12 (0.09)	13,511 (96.81)	279 (2.0)	154 (1.1)	7.23	97.98	52.60	4.12	98.87	3.57	0.95
4.5%	8 (0.06)	13,606 (97.49)	184 (1.32)	158 (1.13)	4.82	98.67	51.74	4.17	98.85	3.61	0.96
5%	5 (0.04)	13,683 (98.04)	107 (0.77)	161 (1.15)	3.01	99.22	51.12	4.46	98.84	3.88	0.98
Comorbid SUD	24 (0.17)	12,961 (92.87)	829 (5.94)	142 (1.02)	14.46	93.99	54.22	2.81	98.92	2.40	0.91
MSHR	121 (0.87)	2783 (19.94)	11,007 (78.87)	45 (0.32)	72.89	20.18	46.54	1.09	98.41	0.91	1.34

Abbreviations: SUD=substance use disorder, MSHR=Manchester Self Harm Rule, TP=true positive, TN=true negative, FP=false positive, FN=false negative, Sens%=sensitivity%, Spec%=specificity%, BAC%=balanced accuracy, PPV%=positive predictive value%, NPV%=negative predictive value%, LR+=positive likelihood ratio, LR-=negative likelihood ratio.

Supplementary Table 4. Classification performances for the prediction of 10-year mortality across MIRACLE-FEP predictions with a range of threshold levels, comorbid SUD, and Manchester Self Harm Rule in the Swedish sample (N=11,504).

**Predictor**	**TP (%)**	**TN (%)**	**FP (%)**	**FN (%)**	**Sens%**	**Spec%**	**BAC%**	**PPV%**	**NPV%**	**LR+**	**LR-**
MIRACLE-FEP											
1.5%	358 (3.11)	760 (6.61)	10380 (90.23)	6 (0.05)	98.35	6.82	52.59	3.33	99.22	1.06	0.24
2%	330 (2.87)	2778 (24.15)	8362 (72.69)	34 (0.3)	90.66	24.94	57.80	3.80	98.79	1.21	0.37
2.5%	300 (2.61)	4816 (41.86)	6324 (54.97)	64 (0.56)	82.42	43.23	62.82	4.53	98.69	1.45	0.41
3%	277 (2.41)	6087 (52.91)	5053 (43.92)	87 (0.76)	76.10	54.64	65.37	5.20	98.59	1.68	0.44
3.5%	256 (2.23)	6824 (59.32)	4316 (37.52)	108 (0.94)	70.33	61.26	65.79	5.60	98.44	1.82	0.48
4%	196 (1.7)	8334 (72.44)	2806 (24.39)	168 (1.46)	53.85	74.81	64.33	6.53	98.02	2.14	0.62
4.5%	165 (1.43)	9087 (78.99)	2053 (17.85)	199 (1.73)	45.33	81.57	63.45	7.44	97.86	2.46	0.67
5%	153 (1.33)	9290 (80.75)	1850 (16.08)	211 (1.83)	42.03	83.39	62.71	7.64	97.78	2.53	0.70
7.5%	106 (0.92)	10155 (88.27)	985 (8.56)	258 (2.24)	29.12	91.16	60.14	9.72	97.52	3.29	0.78
10%	79 (0.69)	10495 (91.23)	645 (5.61)	285 (2.48)	21.70	94.21	57.96	10.91	97.36	3.75	0.83
15%	43 (0.37)	10872 (94.51)	268 (2.33)	321 (2.79)	11.81	97.59	54.70	13.83	97.13	4.91	0.90
20%	21 (0.18)	11045 (96.01)	95 (0.83)	343 (2.98)	5.77	99.15	52.46	18.10	96.99	6.77	0.95
Comorbid SUD	82 (0.71)	10265 (89.23)	875 (7.61)	282 (2.45)	22.53	92.15	57.34	8.57	97.33	2.87	0.84
MSHR	342 (2.97)	1252 (10.88)	9888 (85.95)	22 (0.19)	93.96	11.24	52.60	3.34	98.27	1.06	0.54

Abbreviations: SUD=substance use disorder, MSHR=Manchester Self Harm Rule, TP=true positive, TN=true negative, FP=false positive, FN=false negative, Sens%=sensitivity%, Spec%=specificity%, BAC%=balanced accuracy, PPV%=positive predictive value%, NPV%=negative predictive value%, LR+=positive likelihood ratio, LR-=negative likelihood ratio.

Supplementary Table 5. Classification performances for the prediction of 10-year mortality across MIRACLE-FEP predictions with a range of threshold levels, comorbid SUD, and Manchester Self Harm Rule in the Finnish sample.

Predictor	TP (%)	TN (%)	FP (%)	FN (%)	Sens%	Spec%	BAC%	PPV%	NPV%	LR+	LR-
MIRACLE-FEP											
1.5%	429 (5.87)	265 (3.63)	6607 (90.44)	4 (0.05)	99.08	3.86	51.47	6.10	98.51	1.03	0.24
2%	409 (5.6)	1200 (16.43)	5672 (77.65)	24 (0.33)	94.46	17.46	55.96	6.73	98.04	1.14	0.32
2.5%	385 (5.27)	2311 (31.64)	4561 (62.44)	48 (0.66)	88.91	33.63	61.27	7.78	97.97	1.34	0.33
3%	365 (5.0)	2987 (40.89)	3885 (53.18)	68 (0.93)	84.30	43.47	63.88	8.59	97.77	1.49	0.36
3.5%	349 (4.78)	3422 (46.84)	3450 (47.23)	84 (1.15)	80.60	49.80	65.20	9.19	97.60	1.61	0.39
4%	276 (3.78)	4561 (62.44)	2311 (31.64)	157 (2.15)	63.74	66.37	65.06	10.67	96.67	1.90	0.55
4.5%	211 (2.89)	5187 (71.01)	1685 (23.07)	222 (3.04)	48.73	75.48	62.11	11.13	95.90	1.99	0.68
5%	199 (2.72)	5382 (73.68)	1490 (20.4)	234 (3.2)	45.96	78.32	62.14	11.78	95.83	2.12	0.69
7.5%	127 (1.74)	6263 (85.74)	609 (8.34)	306 (4.19)	29.33	91.14	60.23	17.26	95.34	3.31	0.78
10%	92 (1.26)	6508 (89.09)	364 (4.98)	341 (4.67)	21.25	94.70	57.98	20.18	95.02	4.01	0.83
15%	51 (0.7)	6732 (92.16)	140 (1.92)	382 (5.23)	11.78	97.96	54.87	26.70	94.63	5.78	0.90
20%	16 (0.22)	6841 (93.65)	31 (0.42)	417 (5.71)	3.70	99.55	51.62	34.04	94.25	8.19	0.97
Comorbid SUD	95 (1.3)	6417 (87.84)	455 (6.23)	338 (4.63)	21.94	93.38	57.66	17.27	95.00	3.31	0.84
MSHR	334 (4.57)	1481 (20.27)	5391 (73.8)	99 (1.36)	77.14	21.55	49.34	5.83	93.73	0.98	1.06

Abbreviations: SUD=substance use disorder, MSHR=Manchester Self Harm Rule, TP=true positive, TN=true negative, FP=false positive, FN=false negative, Sens%=sensitivity%, Spec%=specificity%, BAC%=balanced accuracy, PPV%=positive predictive value%, NPV%=negative predictive value%, LR+=positive likelihood ratio, LR-=negative likelihood ratio.

Supplementary Table 6. The fairness analysis for the MIRACLE-FEP 10-year predictions in the Swedish cohort. The table shows the comparison of the AUROC, Brier Score, Slope, and Intercept for immigration status, gender, and socioeconomic status.

Variable	AUROC (95%CI)	Statistical Testing (P-value)	Brier Score (95%CI)	Statistical Testing (P-value)	Slope (95%CI)	Statistical Testing (P-value)	Intercept (95%CI)	Statistical Testing (P-value)
Immigration								
Born in Sweden (N=10170)	0.71 (0.68-0.74)	0.603	0.03 (0.03-0.03)	0.993	0.96 (0.84-1.09)	0.844	-0.30 (-0.69-0.08)	0.666
Born Elsewhere (N=1334)	0.68 (0.58-0.78)		0.02 (0.02-0.03)		0.99 (0.54-1.48)		-0.55 (-2.11-0.74)	
Gender								
Females (N=7668)	0.67 (0.62-0.71)	0.349	0.02 (0.02-0.02)	0.960	0.95 (0.75-1.16)	0.224	-0.58 (-1.27-0.09)	0.933
Males (N=3836)	0.64 (0.60-0.68)		0.05 (0.05-0.06)		0.79 (0.60-0.96)		-0.61 (-1.12--0.14)	
Socioeconomic Status								
Above Median (N=5743)	0.70 (0.66-0.74)	0.680	0.03 (0.03-0.03)	0.998	1.01 (0.83-1.20)	0.472	-0.28 (-0.88-0.28)	0.875
Below Median (N=5751)	0.71 (0.68-0.75)		0.03 (0.03-0.04)		0.93 (0.78-1.09)		-0.34 (-0.85-0.12)	

Supplementary Table 7. The fairness analysis for the MIRACLE-FEP 10y predictions in the Finnish cohort. The table shows the comparison of the AUROC, Brier Score, Slope, and Intercept for gender and socioeconomic status.

**Variable**	**AUROC (95%CI)**	**Statistical Testing (P-value)**	**Brier Score (95%CI)**	**Statistical Testing (P-value)**	**Slope (95%CI)**	**Statistical Testing (P-value)**	**Intercept (95%CI)**	**Statistical Testing (P-value)**
Gender								
Females (N=4028)	0.65 (0.60-0.70)	0.667	0.03 (0.03-0.04)	0.954	0.86 (0.60-1.09)	0.477	-0.43 (-1.34-0.34)	0.067
Males (N=3277)	0.66 (0.63-0.69)		0.08 (0.07-0.09)		0.94 (0.76-1.13)		0.28 (-0.19-0.75)	
Socioeconomic Status								
Above Median (N=3653)	0.66 (0.62-0.70)	0.006	0.04 (0.04-0.05)	0.969	0.92 (0.70-1.16)	0.294	-0.24 (-0.95-0.40)	0.019
Below Median (N=3652)	0.73 (0.70-0.76)		0.07 (0.06-0.08)		1.06 (0.91-1.21)		0.67 (0.23-1.10)	

Supplementary Table 8. First-line (i.e., within 30 days post-FEBD) medication use stratified by MIRACLE-FEP predictions in the Swedish cohort.

MIRACLE-FEP Risk Prediction Category	N of Users	% of Users (95%CI)	Odds Ratio (95%CI)
Use of Antipsychotics			
MIRACLE-FEP:<1% (N=20,329)	4345	21.4 (20.8-21.9)	1 (reference)
MIRACLE-FEP:1-2% (N=7914)	2388	30.2 (29.1-31.2)	1.59 (1.50-1.69)
MIRACLE-FEP:2-5% (N=2296)	807	35.1 (33.1-37.0)	1.99 (1.82-2.19)
MIRACLE-FEP:>5% (N=474)	177	37.3 (32.9-41.8)	2.19 (1.81-2.65)
Use of Mood Stabilizers			
MIRACLE-FEP:<1% (N=20,329)	9873	48.6 (47.9-49.2)	1 (reference)
MIRACLE-FEP:1-2% (N=7914)	3843	48.6 (47.5-49.6)	1.00 (0.95-1.05)
MIRACLE-FEP:2-5% (N=2296)	1083	47.2 (45.1-49.1)	0.95 (0.87-1.03)
MIRACLE-FEP:>5% (N=474)	215	45.4 (41.4-50.0)	0.88 (0.73-1.06)
Use of Antipsychotic + Mood Stabilizer			
MIRACLE-FEP:<1% (N=20,329)	1873	9.2 (8.8-9.6)	1 (reference)
MIRACLE-FEP:1-2% (N=7914)	1117	14.1 (13.3-14.8)	1.62 (1.50-1.75)
MIRACLE-FEP:2-5% (N=2296)	374	16.3 (14.9-17.8)	1.92 (1.70-2.16)
MIRACLE-FEP:>5% (N=474)	84	17.7 (14.3-21.3)	2.12 (1.66-2.68)
Use of Antidepressants			
MIRACLE-FEP:<1% (N=20,329)	11,002	54.1 (53.4-54.8)	1 (reference)
MIRACLE-FEP:1-2% (N=7914)	4526	57.2 (56.2-58.3)	1.13 (1.07-1.19)
MIRACLE-FEP:2-5% (N=2296)	1366	59.5 (57.5-61.6)	1.25 (1.14-1.36)
MIRACLE-FEP:>5% (N=474)	255	53.8 (49.2-58.2)	0.99 (0.82-1.19)
Use of Benzodiazepines and Related			
MIRACLE-FEP:<1% (N=20,329)	2305	11.3 (10.9-11.7)	1 (reference)
MIRACLE-FEP:1-2% (N=7914)	1413	17.9 (17.1-18.7)	1.70 (1.58-1.83)
MIRACLE-FEP:2-5% (N=2296)	417	18.2 (16.5-19.8)	1.74 (1.55-1.94)
MIRACLE-FEP:>5% (N=474)	110	23.2 (19.6-26.8)	2.36 (1.89-2.93)

Supplementary Table 9. First-line (i.e., within 30 days post-FEBD) medication use stratified by MIRACLE-FEP predictions in the Finnish cohort.

MIRACLE-FEP Risk Prediction Category	N of Users	% of Users (95%CI)	Odds Ratio (95%CI)
Use of Antipsychotics			
MIRACLE-FEP:<1% (N=8269)	1812	21.9 (21.1-22.8)	1 (reference)
MIRACLE-FEP:1-2% (N=4571)	1165	25.5 (24.3-26.8)	1.22 (1.12-1.33)
MIRACLE-FEP:2-5% (N=1004)	242	24.1 (21.6-26.9)	1.13 (0.97-1.32)
MIRACLE-FEP:>5% (N=112)	22	19.6 (12.5-27.7)	0.87 (0.53-1.37)
Use of Mood Stabilizers			
MIRACLE-FEP:<1% (N=8269)	1791	21.7 (20.8-22.6)	1 (reference)
MIRACLE-FEP:1-2% (N=4571)	1274	27.9 (26.6-29.1)	1.40 (1.29-1.52)
MIRACLE-FEP:2-5% (N=1004)	336	33.5 (30.6-36.3)	1.82 (1.58-2.09)
MIRACLE-FEP:>5% (N=112)	37	33.0 (25.0-42.0)	1.78 (1.19-2.64)
Use of Antipsychotic + Mood Stabilizer			
MIRACLE-FEP:<1% (N=8269)	484	5.9 (5.4-6.3)	1 (reference)
MIRACLE-FEP:1-2% (N=4571)	474	10.4 (9.5-11.3)	1.86 (1.63-2.12)
MIRACLE-FEP:2-5% (N=1004)	124	12.4 (10.4-14.4)	2.27 (1.83-2.79)
MIRACLE-FEP:>5% (N=112)	14	12.5 (6.3-18.8)	2.30 (1.25-3.92)
Use of Antidepressants			
MIRACLE-FEP:<1% (N=8269)	4017	48.6 (47.5-49.7)	1 (reference)
MIRACLE-FEP:1-2% (N=4571)	2058	45.0 (43.6-46.5)	0.87 (0.81-0.93)
MIRACLE-FEP:2-5% (N=1004)	487	48.5 (45.5-51.5)	1.00 (0.87-1.14)
MIRACLE-FEP:>5% (N=112)	57	50.9 (42.0-59.8)	1.10 (0.76-1.60)
Use of Benzodiazepines and Related			
MIRACLE-FEP:<1% (N=8269)	801	9.7 (9.0-10.3)	1 (reference)
MIRACLE-FEP:1-2% (N=4571)	628	13.7 (12.8-14.8)	1.48 (1.33-1.66)
MIRACLE-FEP:2-5% (N=1004)	172	17.1 (14.8-19.6)	1.93 (1.61-2.30)
MIRACLE-FEP:>5% (N=112)	20	17.9 (10.7-25.0)	2.03 (1.21-3.23)

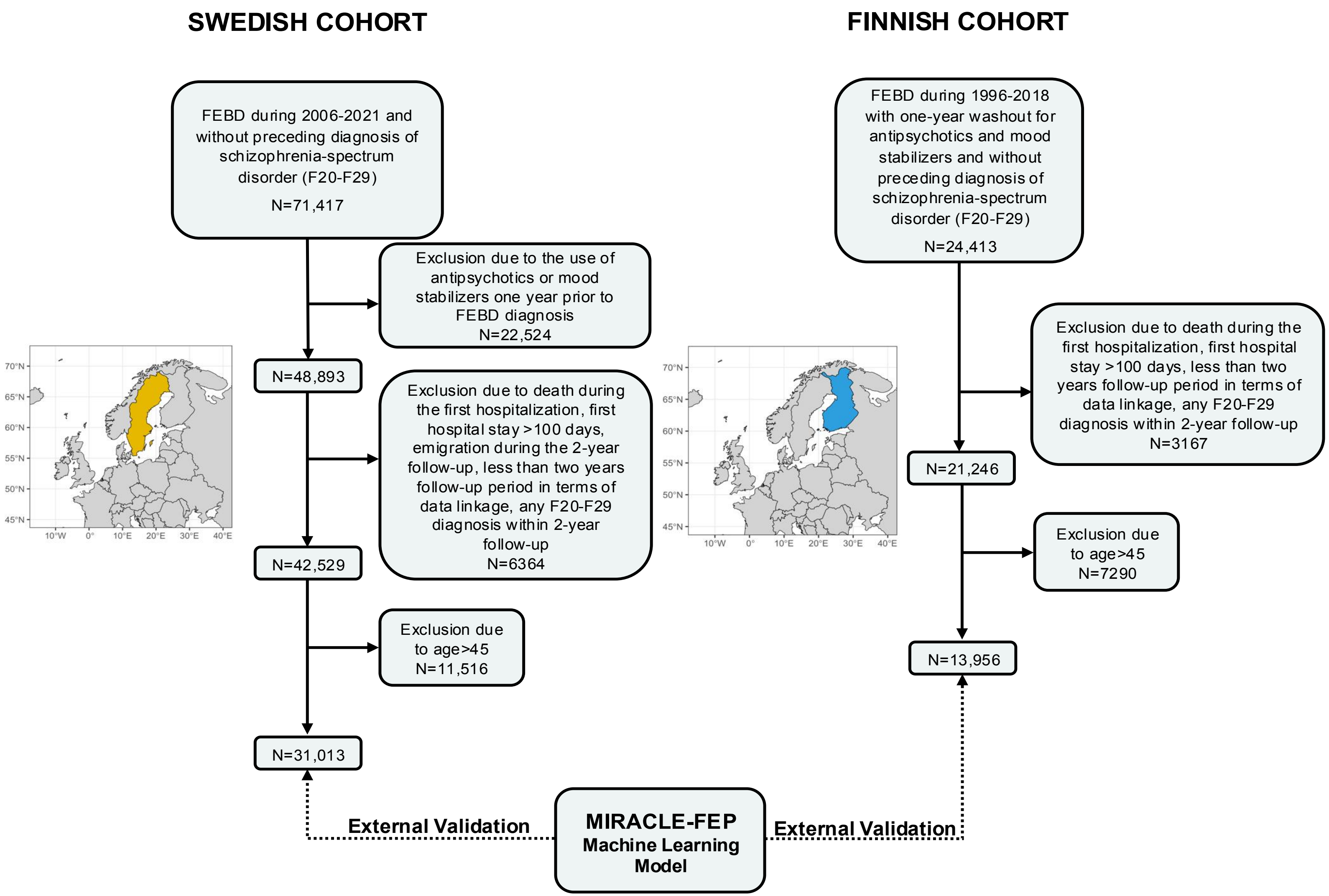

Supplementary Figure 1. A flowchart depicts the Swedish and Finnish cohorts and the analyses of the present study.

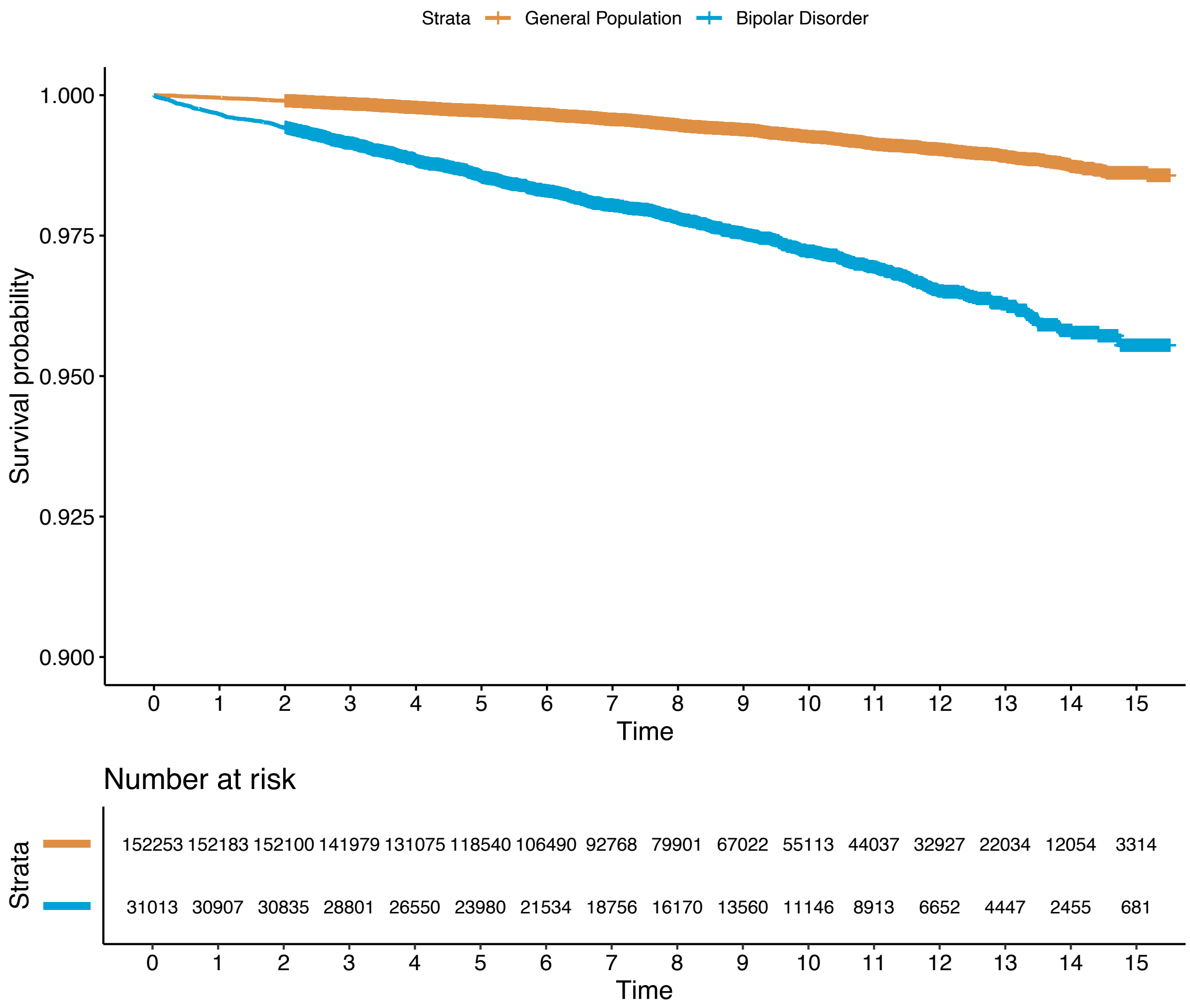

Supplementary Figure 2. Kaplan-Meier curves showing survival in the Swedish first-episode bipolar disorder (FEBD) cohort (N=31,013) compared to an age- and sex-matched sample from the general population sample (N=152,253) over the available follow-up.

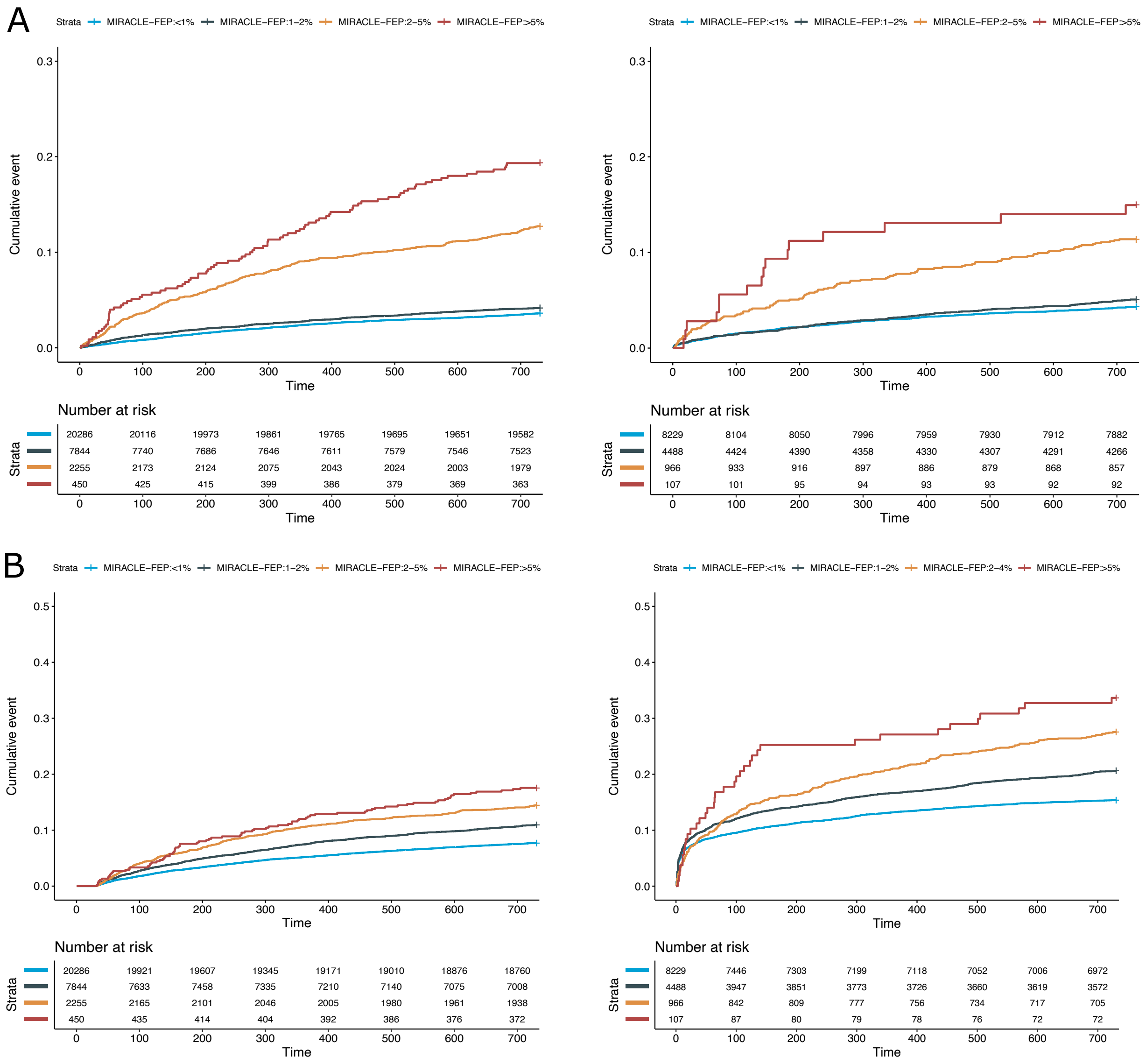

Supplementary Figure 3. A) Cumulative incidence of hospitalization due to a suicide attempt among two-year survivors stratified by the MIRACLE-FEP predictions in the Swedish (left) and the Finnish (right) cohorts. B) Cumulative incidence of rehospitalization due to bipolar disorder among two-year survivors stratified by the MIRACLE-FEP predictions in the Swedish (left) and the Finnish (right) cohorts.

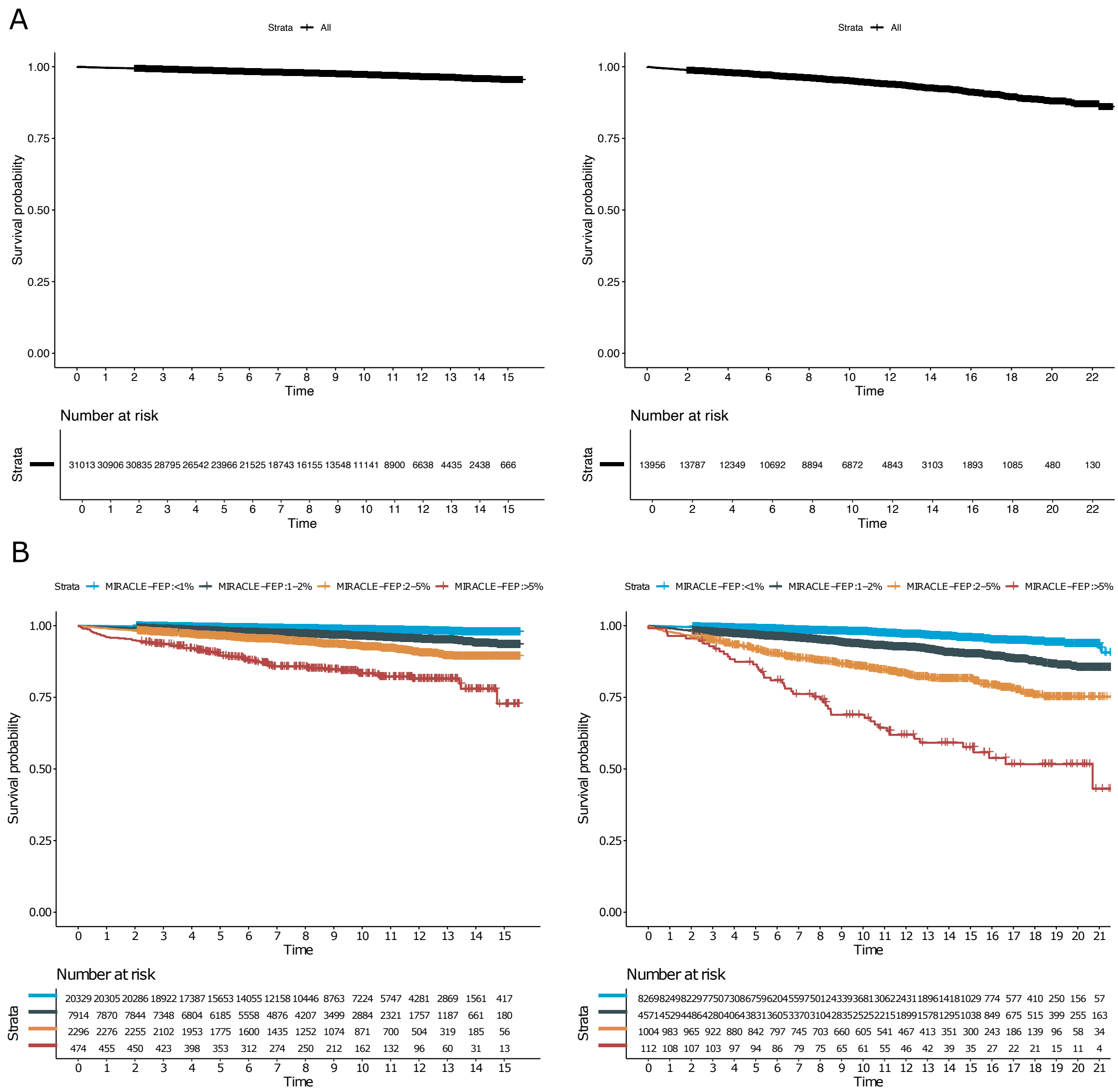

Supplementary Figure 4. A) Kaplan-Meier plots for survival in the Swedish (left) and the Finnish (right) cohorts with total follow-up (time in years) and censoring without stratification. B) Kaplan-Meier plots for survival in the Swedish (left) and the Finnish (right) cohorts with total follow-up (time in years) and censoring with stratification using MIRACLE-FEP predictions. Log-rank tests, P-value<0.0001 in both the Swedish and the Finnish cohorts.

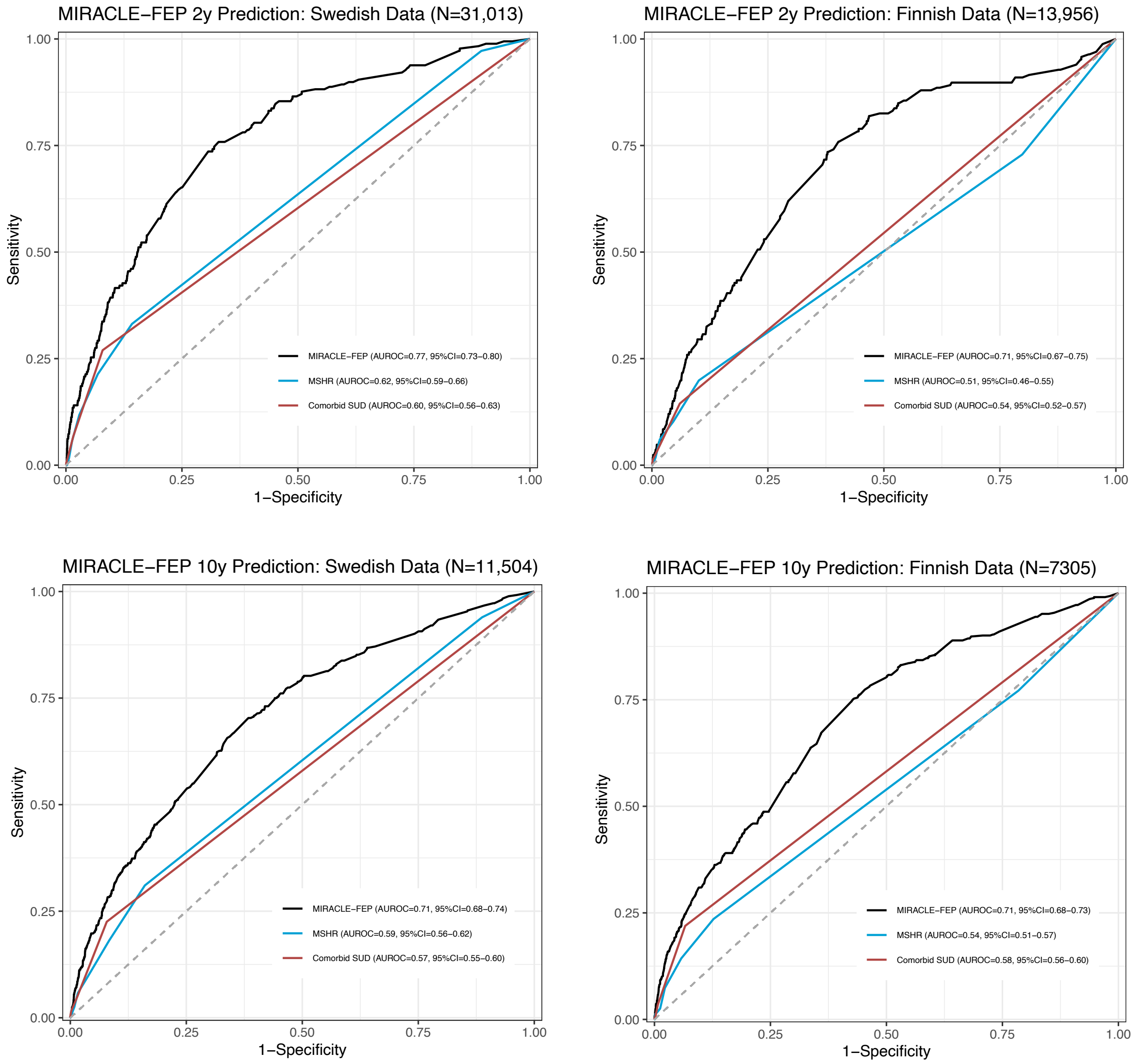

Supplementary Figure 5. The MIRACLE-FEP model's predictions for two-year and ten-year mortality compared with the predictions made by the Manchester Self-Harm Rule (MSHR) and the presence of comorbid substance use disorder (SUD).

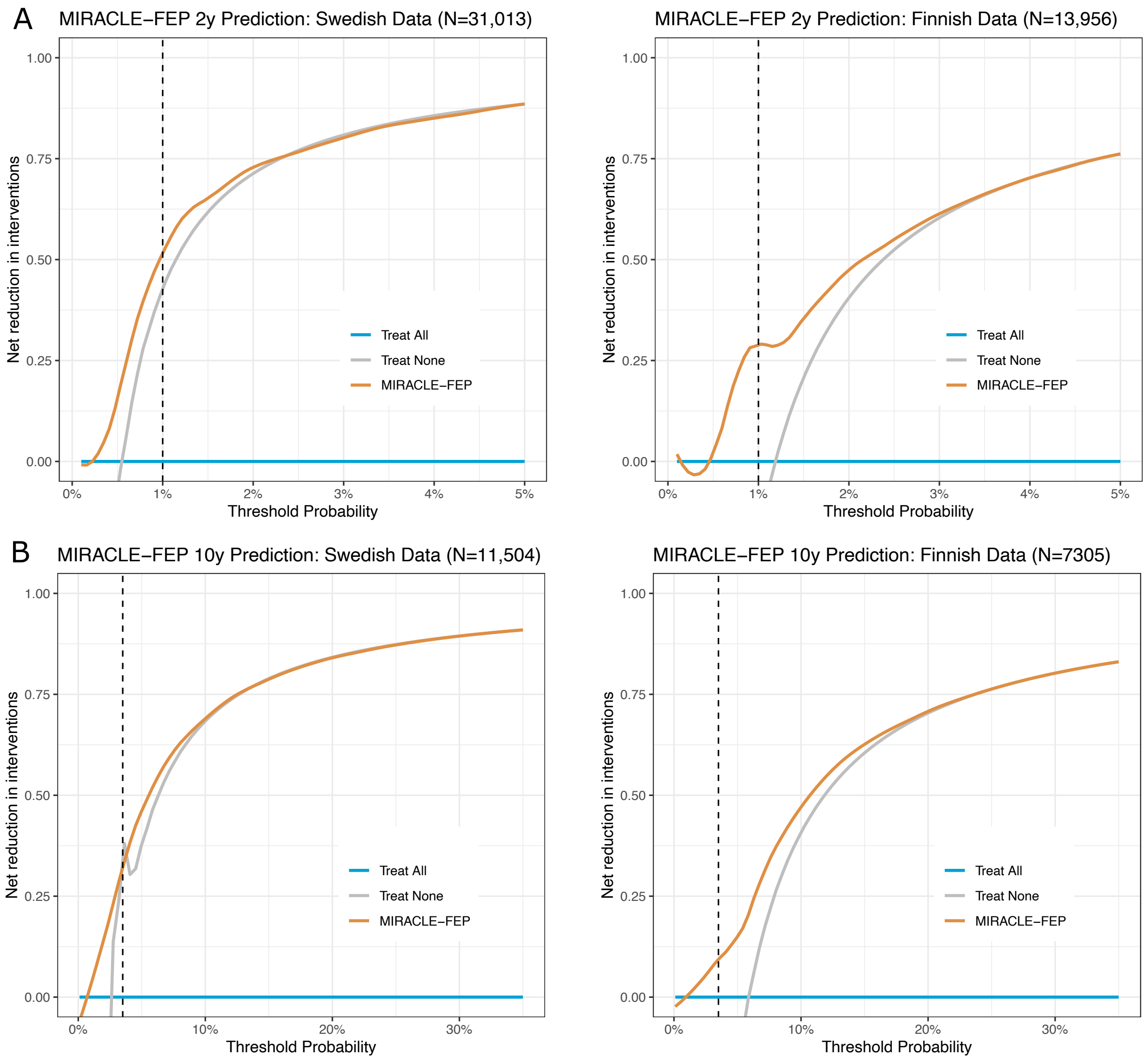

Supplementary Figure 6. Decision curve analysis for a net reduction in the number of people needing intensive treatment or monitoring if used MIRACLE-FEP for decision making for A) a two-year and B) 10 years post-FEBD. The dashed line presents the estimated mortality.

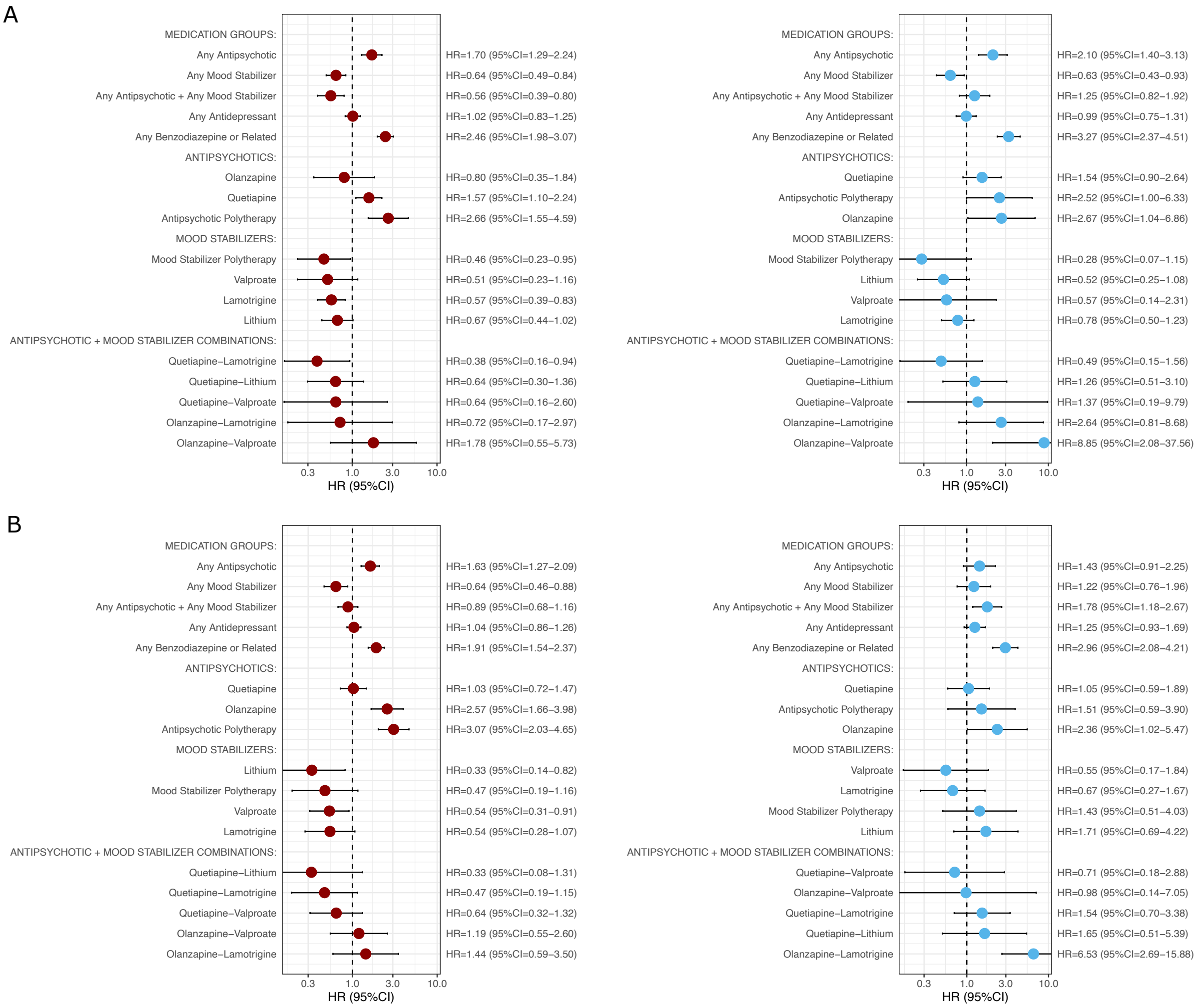

Supplementary Figure 7. Association of different pharmacotherapies and the risk of death in the A) Swedish cohort with up to 15 years of follow-up and B) the Finnish cohort with up to 23 years of follow-up. Both cohorts are stratified based on two-year MIRACLE-FEP predictions, using a 1% cutoff (red for individuals above and blue for those below this threshold). Each pharmacotherapy was compared with its non-use, except for antipsychotics and mood stabilizers, which were each compared with the non-use of either antipsychotics or mood stabilizers.

Supplementary References

1. Poranen J, Koistinaho A, Tanskanen A, Tiihonen J, Taipale H, Lähteenvuo M. Twenty-year medication use trends in first-episode bipolar disorder. *Acta Psychiatr Scand*. 2022;146(6):583-593. doi:10.1111/acps.13504

2. Koistinaho A, Poranen J, Tanskanen A, Tiihonen J, Taipale H, Lähteenvuo M. Real-world use of pharmacological treatments for incident bipolar disorder: A Finnish nationwide cohort study. *J Affect Disord*. 2023;340:237-244. doi:10.1016/j.jad.2023.08.015

3. Archer L, Snell KIE, Ensor J, Hudda MT, Collins GS, Riley RD. Minimum sample size for external validation of a clinical prediction model with a continuous outcome. *Statistics in Medicine*. 2021;40(1):133-146. doi:10.1002/sim.8766

4. Riley RD, Debray TPA, Collins GS, et al. Minimum sample size for external validation of a clinical prediction model with a binary outcome. *Statistics in Medicine*. 2021;40(19):4230-4251. doi:10.1002/sim.9025

5. Lieslehto J, Tiihonen J, Lähteenvuo M, et al. Development and Validation of a Machine Learning–Based Model of Mortality Risk in First-Episode Psychosis. *JAMA Network Open*. 2024;7(3):e240640-e240640. doi:10.1001/jamanetworkopen.2024.0640
